# Characterization of extracellular vesicles in COVID-19 infection during pregnancy

**DOI:** 10.3389/fcell.2023.1135821

**Published:** 2023-07-25

**Authors:** Ayelet Dangot, Mor Zavaro, Tali Hana Bar-Lev, Lian Bannon, Ayala Zilberman, Eliana Pickholz, Irit Avivi, Anat Aharon

**Affiliations:** ^1^ Hematology Research Laboratory, Tel-Aviv Sourasky Medical Center, Tel Aviv, Israel; ^2^ Obstetrics and Gynecology Department, Lis Hospital for Women, Tel Aviv Sourasky Medical Center, Tel Aviv, Israel; ^3^ Sackler Faculty of Medicine, Tel Aviv University, Tel Aviv, Israel; ^4^ Department of Medicine F, Tel-Aviv Sourasky Medical Center, Tel Aviv, Israel; ^5^ Hematology Department, Tel-Aviv Sourasky Medical Center, Tel Aviv, Israel

**Keywords:** SARS-CoV-2, pregnancy, extracellular vesicles (EVs), cytokine, coagulation

## Abstract

**Introduction:** SARS-CoV-2 infection may cause a severe inflammatory response, inflicting severe morbidity and mortality. This risk is modestly increased in pregnant patients. Despite the hypercoagulability and immunosuppression associated with pregnancy, most pregnant women experience a mild COVID-19 infection. Maternal extracellular vesicles (EVs) may interact with endothelial and immune components to facilitate a favorable disease course. This pilot study aimed to explore the characteristics of EVs released during COVID-19 infection occurring during the third trimester of pregnancy.

**Methods:** In this prospective study, blood samples were obtained from 16 healthy non-pregnant (NP), 18 healthy-pregnant (HP), and 22 COVID-19 positive pregnant subjects (CoV-P). Disease course and pregnancy outcomes were assessed and EVs were characterized. Of note, limited volumes of sample acquired from the subjects made it necessary to use smaller and different subsets of samples for each analysis.

**Results:** The majority (91%) of the COVID-19-pregnant subjects (18 mild and 2 moderate disease) experienced good pregnancy-related outcomes. EV concentrations were higher in healthy-pregnant subjects compared to non-pregnant subjects (*p* = 0.0041) and lower in COVID-19-pregnant subjects compared to healthy-pregnant subjects (*p* = 0.0150). CD63 exosome marker expression was higher in EVs of healthy-pregnant subjects and COVID-19-pregnant subjects compared to EVs of non-pregnant subjects (*p* = 0.0149, *p* = 0.0028, respectively). Similar levels of SARS-CoV-2 entry proteins (ACE-2 and TMPRSS2) were found in all three groups. Cytokine content increased in healthy-pregnant subject-EVs compared to non-pregnant EVs, while IL-2 and IL-6 levels were decreased in COVID-19-pregnant subject-EVs compared to healthy-pregnant subject-EVs (*p* = 0.043, *p* = 0.0390, respectively). CD8^+^, cytotoxic T-cell marker, was lower in non-pregnant EVs compared to healthy-pregnant subject-EVs and to COVID-19-pregnant subjects (*p* = 0.0108, *p* < 0.0001, respectively). COVID-19- pregnant subject-EVs demonstrated higher levels of platelet activation marker (CD62P) than non-pregnant (*p* = 0.0327) and healthy-pregnant subjects (*p* = 0.0365). Endothelial marker EV-CD144+ was lower in healthy-pregnant subjects versus non-pregnant subjects (*p* = 0.0093), but similar in COVID-19-pregnant and non-pregnant subjects. Other EVs’ coagulation markers/activity, D-Dimer and fibrinogen levels were similar in healthy-pregnant subjects and COVID-19 positive pregnant subjects.

**Conclusion:** COVID-19 positive pregnant subjects’ EVs demonstrated an attenuated inflammatory response, with no additional activation of the coagulation system.

## 1 Introduction

SARS-CoV-2 infection during pregnancy initially raised concern over possible adverse maternal outcomes. Later studies have shown that the majority of pregnant women experience mild disease (Overton et al., 2022). Although SARS-CoV-2 infection during pregnancy is associated with a modest increase in risk of morbidity and pregnancy related complications (Ciapponi et al., 2021; Rajput and Sharma, 2021; Male, 2022), these complications are mostly restricted to patients with severe disease (Rajput and Sharma, 2021; Male, 2022). These patients are at increased risk of cesarean delivery and preterm birth (Marchand et al., 2022), and the risk increases further in those with severe disease and pre-existing risk factors such as older age, obesity and gestational diabetes (Conde-Agudelo and Romero, 2022).

Despite the immunomodulation and increased thrombogenicity associated with pregnancy (Silasi et al., 2015), the incidence of severe cytokine storm and hypercoagulability-related complications seems to be confined only to severe cases. However, most pregnant women display a mild inflammatory response to SARS-CoV-2 infection (Garcia-Flores et al., 2022) and recover without requiring hospitalization (Metz et al., 2022).

SARS-CoV-2 infection may trigger an inflammatory response (Costela-Ruiz et al., 2020). Extensive vascular inflammation injures the tissues, causing them to release cytokines such as IL-6, IL-1β, and TNF-α, triggering endothelial exocytosis and accelerating vascular injury (Lowenstein and Solomon, 2020; Gustine and Jones, 2021). SARS-CoV-2 binds to cells through angiotensin-converting enzyme 2 (ACE2) receptors (Yeung et al., 2021). Transmembrane serine protease 2 (TMPRSS2), a protein that cleaves ACE2, facilitating entry of coronaviruses into their target cells (Stopsack et al., 2020). Pro-inflammatory cytokines induce shedding of soluble ACE2 which may reduce SARS-CoV-2 entry into cells but increases the activation of the AngII/AT1R axis that accelerates the inflammation response (Scialo et al., 2020). The inflammatory response may also trigger various coagulation abnormalities, inducing the hypercoagulable state responsible for severe COVID-19 complications such as thrombotic microangiopathy and venous thromboembolism (VTE) (Jacob et al., 2020; Kichloo et al., 2020). There are several proposed mechanisms for the hypercoagulable state in COVID-19 patients. These include endothelial injury, elevation in circulating tissue factor (TF), and activation of the coagulation cascade. Another proposed mechanism is induction of thrombosis as a response to elevation in levels of pro-inflammatory cytokines (Kichloo et al., 2020). This hyper inflammatory state, combined with neutrophil accumulation, platelet activation, and EV aggregates, induces thrombosis (Caillon et al., 2022).

Extracellular vesicles (EVs) are sub-micron membrane vesicles. The population of EVs include small EVs (size <100 nm, part of them are exosomes) and large EVs (size 100–1,000 nm) (Thery et al., 2018) that bud from cell membranes under various conditions (Teng and Fussenegger, 2020). EVs bear antigens reflecting their cellular origins, and interact with target cells by transferring their contents, including cytokines, DNA, RNA and microRNAs (Teng and Fussenegger, 2020). EVs may reflect disease status and severity, as we have previously demonstrated in diabetic foot ulcer patients (Tsimerman et al., 2011), Alzheimer’s disease (Aharon et al., 2020) and β-thalassemia (Levin et al., 2018). EVs have been shown to potentiate the pro-coagulation pathway during SARS-CoV-2 infection (Balbi et al., 2021). Moreover, previous studies have demonstrated that the contents within EVs reflect disease status in COVID-19 patients during infection (Krishnamachary et al., 2021). Several studies demonstrated the role of EVs in the cytokine storm, as they accelerate vascular and tissue injury (Pillalamarri et al., 2021) during SARS-CoV-2 infection (Gurunathan et al., 2021; Caillon et al., 2022). Expression of activated platelet markers was elevated in EVs of subjects with non-severe COVID-19 infection compared to severe COVID-19 patients (Zaid et al., 2020). Moreover EVs, unlike patients’ coagulation profiles, reflected COVID-19 patients disease severity. EVs obtained from patients with moderate and severe disease display elevated levels of immune and vascular-related markers (Aharon A. et al., 2023).

Placental EVs are released into the maternal blood stream and can be detected in maternal circulation throughout pregnancy (Ortega et al., 2022). Placental EVs interact with endothelial and immune components and may contribute to systemic inflammation. However, they also downregulate T-cell activity and may contribute to fetal allograft immune escape (Germain et al., 2007; Toth et al., 2007). Placental EVs may influence disease course in COVID-19 patients infected during pregnancy.

This study aimed to characterize the EV population in women infected with SARS-CoV-2 during pregnancy and detect similarities and differences in EVs of healthy-pregnant subjects compared to subjects infected with COVID-19 during pregnancy.

## 2 Methods

### 2.1 Study population

This prospective study was conducted at the Tel Aviv Sourasky Medical Center in Tel Aviv, Israel between July 2020 and April 2021, during the second and third waves of the COVID-19 pandemic, which were dominated by the SARS-CoV-2 alpha and delta variants. The study was approved by the local IRB according to the Helsinki principles (Approval No 0759-19). The study population consisted of 22 SARS-CoV-2 positive pregnant subjects (CoV-P) in their third trimesters of pregnancy (mean gestational weeks 36, IQR [28.5, 38]). The control groups included 16 healthy female, non-pregnant subjects (NP) matched by age, that were recruited from the hospital’s staff and 18 healthy-pregnant subjects (HP) at term (mean gestational week 39, IQR [39, 40]) matched by age. The HP subjects were recruited after 39 weeks of gestation when they underwent a routine check at the hospital. CoV-P subjects were recruited upon arrival to the hospital due to symptoms or obstetric indication. Subjects in active labor were excluded. COVID-19 status was confirmed by a positive nucleic acid RT-PCR at admission or within the 10 days prior to enrollment. Seven of the CoV-P subjects were vaccinated (32%) with two doses of the BioNTech, Pfizer vaccine. Two CoV-P subjects (9%) had prior SARS-CoV-2 infection, over 9 months prior to enrollment. All controls (NP and HP) were vaccinated with two doses of the BioNTech, Pfizer vaccine, and were recruited up to 6 months after the second dose. Blood samples were obtained at enrollment after receiving informed consent.

### 2.2 Blood tests

Routine blood count and coagulation tests were performed. All laboratory tests are detailed in Table 1.

**TABLE 1:** Study cohort characteristics.

Variable	NP = 16	HP = 18	CoV-P = 22	*p*-value NP vs. HP	*p*-value NP vs. CoV-P	*p*-value HP vs. CoV-P	ANOVA
Age (years)	36.5 [31,41],	33 [30.5,36.5]	32 [27.5,36.5]	0.1288	0.0667	0.5316	0.251
BMI	24.455 ± 3.1277 *n = 15	23.842 ± 2.986 *n = 16	23.518 ± 3.760 *n = 19	0.5815	0.4440	0.7824	0.7187
Vaccinated	16 (100%)	18 (100%)	7 (32%)	1	**<0.0001**	**<0.0001**	**<0.0001**
Gravidity (number pregnancies)	1.5 [0,3]	2 [1,2]	3 [1,5]	0.4265	0.0375	0.1257	0.135
Parity (number of deliveries)	1.5 [0,1.5]	0 [0,1]	2 [0,2]	0.2293	0.6073	0.0524	0.205
Gestational week	N/A	39 [39, 40]	36 [28.5,38]	N/A	N/A	**<0.001**	**0.001**
**Comorbidities**							
Asthma (%)	1 (6.25%)	0	1 (4.54%)	0.288	0.818	0.366	0.788
Hypothyroidism	1 (6.25%)	1(5.5%)	1 (4.54%)	0.927	0.818	0.891	0.997
Systolic blood pressure (mmHg)	112 ± 5.099	115.3 ± 6.03	116.6 ± 11.74	0.2986	0.2356	0.3738	0.524
Diastolic blood pressure (mmHg)	69.25 ± 4.349	67.88 ± 6.84	68.52 ± 8.909	0.6364	1	0.6788	0.961
Heart rate (bpm)	75.2 ± 5.404	82.5 ± 9.716	90.2 ± 22.17	0.1484	0.051	0.1179	0.122
WBC (10^3/mL)	6.57 ± 1.078 *n = 10	9.313 ± 1.198 *n = 16	8.564 ± 2.201	0.0002	0.0082	0.1686	0.001
PLT (10^3/mL)	259.4 ± 63.7 *n = 10	190.9 ± 48.06 *n = 16	168.7 ± 81.86	0.0171	0.0117	0.963	0.009
Fibrinogen (mg/dL)	311.4 ± 54.57 *n = 10	501.3 ± 91.87 *n = 16	527.2 ± 95.56	0.0003	<0.0001	0.5137	<0.0001
D-Dimer (mg/L)	0.356 ± 0.3223*n = 10	1.695 ± 0.731 *n = 14	1.75 ± 0.8593*n = 18	0.0001	<0.0001	0.8942	<0.0001

Categorical variables are presented as median [IQR].

Continuous variables are presented as mean ± STD. All variables were tested for normality by Shapiro-Wilk normality test and did not show normal distribution in all parameters except BMI.

*p*-value for continuous variables calculated via Kruskal- Wallis ANOVA to compare all groups, and Mann-Whitney test to compare two groups.

*p*-value for categorical variables calculated via Chi-Square test.

All other variables displayed as number (percent).

Vaccinated- COVID-19 vaccine, two doses of the BioNTech, Pfizer vaccine.

Assisted delivery-vacuum extraction or emergent cesarean section.

ICU, intensive care unit.

When sample size was lower than mentioned in the top of the table, the exact number appears with *.

### 2.3 EV isolation

EVs were isolated from platelet poor plasma (PPP) in accordance to MISEV 2018 (Thery et al., 2018). Specifically, platelet poor plasma (PPP) was obtained within 1 hour of collection and frozen in aliquots at −80°C (Yuana et al., 2015). Two sequential centrifugations (15 min 1,500× *g*, 24°C) were performed to remove apoptotic bodies and cell debris prior to collection. EV pellets of small and large EVs were isolated from thawed PPP by 1 hour of centrifugation (MIKRO 220R, rotor 1189-A, Hettich 20,000g, 4°C, braking -zero). Previous studies compared EVs characteristics obtained by ultracentrifugation (UC) 20,000 g vs. 100,000 g (Lee et al., 2012; Ettelaie et al., 2014). Specifically, Saari H. at all demonstrated that the size of both populations obtained by 20,000 g or 100,000 g overlapped when measured by NTA (Saari et al., 2015). The importance of the fractions enriched with “large EVs” obtained by 20,000 g was demonstrated in recent studies (Aharon et al., 2021; Aharon A. et al., 2023). Therefore, in the current study EV pellets were isolated by 20,000 g as done in other studies (Xie et al., 2014; Abbaszade Dibavar et al., 2018; Zhang et al., 2018) and used for western blot assay.

### 2.4 EV characteristics

The size, concentration, and membrane antigen expression of EVs were validated on thawed, diluted PPP and pellet EV samples.

#### 2.4.1 EV size and concentration

PPP and pellet EV size and concentration were validated by nanoparticle tracking analysis (NTA; Malvern Panalytical NanoSight NS300). Nanoparticle tracking analysis: EVs were diluted in filtered PBS (1:200) and introduced into the sample chamber using a syringe pump. Five video recordings were made for a period of 30 s each, using NanoSight software with the following settings: detection threshold 5 and camera level 12.

#### 2.4.2 EV proteins content

EV pellet expression of proteins including cytokines, placental marker (hPL), TMPRSS2, ACE2, FOXP3, CD63, and CD81, was tested by western blot (Supplementary Table S1). Briefly, 30 ul of EV pellets obtained from similar PPP volumes (250ul) were combined with a 2xlysis buffer (RayBiotech) supplemented with 1% proteinase inhibitor and 1% phosphatase inhibitors (Sigma) containing β-mercaptoethanol (1:20, Biorad). Samples were loaded and separated on 4%–20% Mini-PROTEAN TGX Precast Protein Gels (Bio-Rad) and then transferred to Trans-Blot Turbo Mini 0.2 μm Nitrocellulose Transfer Packs (Bio-Rad). The membranes were stained with Ponceau S solution (P7170, sigma) to ensure protein transfer from the gel to the membrane. Membranes were washed and immunoblotted. The membranes were incubated with specific antibodies, documented by myECL™ Imager and analyzed by My Image Analysis Software (both from Thermo Fisher Scientific, Waltham, MA United States).

#### 2.4.3 EV membrane antigen expression

PPP EV membrane antigen levels were assessed by flow cytometry (CytoFLEX, Beckman Coulter, United States) using fluorescent antibodies (Supplementary Table S1) according to the MIFlowCyt-EVs standards (Welsh et al., 2020). Specifically, EV gates were set using Megamix, a mix of fluorescent beads (0.5/0.9/3 µm beads; Biocytex, Marseille, France) (Supplementary Figure S4A), a “gold standard” size gate calibration bead mix (Robert et al., 2009; Robert et al., 2012) and 0.2 µm polystyrene beads (Malvern Pananalytical, United Kingdom) (Supplementary Figure S4B). EV analysis included several controls that were used to set the analysis including buffer only, unstained samples, appropriate isotype controls and single-stain antibodies as required (Supplementary methods MIFlowCyt checklist) (Lee et al., 2008). Events were collected by time at a flow rate of 10 µL per minute. Controls and samples were analyzed in the same acquisition setting and reagent conditions. Instrument configuration and settings: Gain: FSC 500; SSC 100; Violet SSC 40; PE 120; APC400; FITC 100, Threshold: manual 10000 height.

#### 2.4.4 EV coagulation activity

EV pellet coagulation activity was validated by the Tissue Factor activity assay kit (Abcam, ab108906). This assay measures the ability of lipoprotein TF/FVIIa to activate factor X (FX) to factor Xa. The amidolytic activity of the TF/FVIIa complex was quantified by the amount of FXa produced using a highly specific FXa substrate releasing a yellow para-nitroaniline (pNA) chromophore. The change in absorbance of the pNA at 405 nm is directly proportional to the TF enzymatic activity.

### 2.5 Statistical analysis

Statistical analysis was performed using the GraphPad Prism 5 software (GraphPad Software Inc., CA, United States). Normal distribution was tested by Shapiro-Wilk normality test. When one or more of the groups did not pass the normality test (alpha = 0.05), a non-parametric test was used for statistics analysis. Results were assessed by Kruskal Wallis and Dunn’s multiple comparison test or one-way analysis of variance (ANOVA) and the Tukey’s multiple comparison test to compare the study groups. When only two groups were compared, nonparametric two-tailed Mann-Whitney *U* test or Student’s t-tests were used. *p* < 0.05 was considered statistically significant. Spearman’s r correlations between pregnancy week and EV membrane antigen expression or protein expression were performed.

## 3 Results

### 3.1 Patient characteristics

Study population characteristics are summarized in Table 1. The mean age, BMI, gravidity, parity and blood pressure were similar between all groups. Mild comorbidities were rare across all groups and included asthma and controlled hypothyroidism. Gravidity and parity ranged between one and six in the different groups, without a significant difference between groups. Gestational week at enrollment was significantly lower in all pregnant subjects with COVID-19, compared to HP.

### 3.2 Disease course and pregnancy outcomes

The 22 CoV-P subjects were categorized according to disease severity, as classified by the WHO (NIH COVID-19 Treatment Guidelines. National Institutes of Health Clinical Spectrum of SARS-CoV-2 Infection. https://www.covid19treatmentguidelines.nih.gov). Eighteen subjects (82%) were classified with asymptomatic to mild disease, two subjects were classified with moderate disease (9%) and two subjects were classified with severe disease (9%). CoV-P subjects with mild or moderate disease did not require oxygen administration, admission to the intensive care unit (ICU) or prolonged hospitalization and were discharged in good condition. The two subjects that presented with severe disease required oxygen supplementation and extended hospital stay. One of these subjects required invasive ventilation and prolonged rehabilitation due to altered neurologic state. The subject was ultimately discharged home with grade 1 cognitive impairment. There was no evidence of VTE events in CoV-P subjects of any disease severity. Preterm delivery occurred in three CoV-P subjects (13.64%), one with mild disease and two with severe disease. No preterm deliveries occurred in HP. Assisted delivery by vacuum extraction or cesarean delivery due to fetal indication (non-reassuring fetal heart rate on fetal monitor) occurred in two HP (12.5%), and three CoV-P subjects (13.64%), two of whom were classified as having mild COVID-19 and one with moderate disease. Cesarean delivery due to maternal indication (respiratory distress or hemodynamic instability) occurred only in two CoV-P subjects (9.1%), both classified with severe COVID-19.

### 3.3 General laboratory tests

**FIGURE 1:**
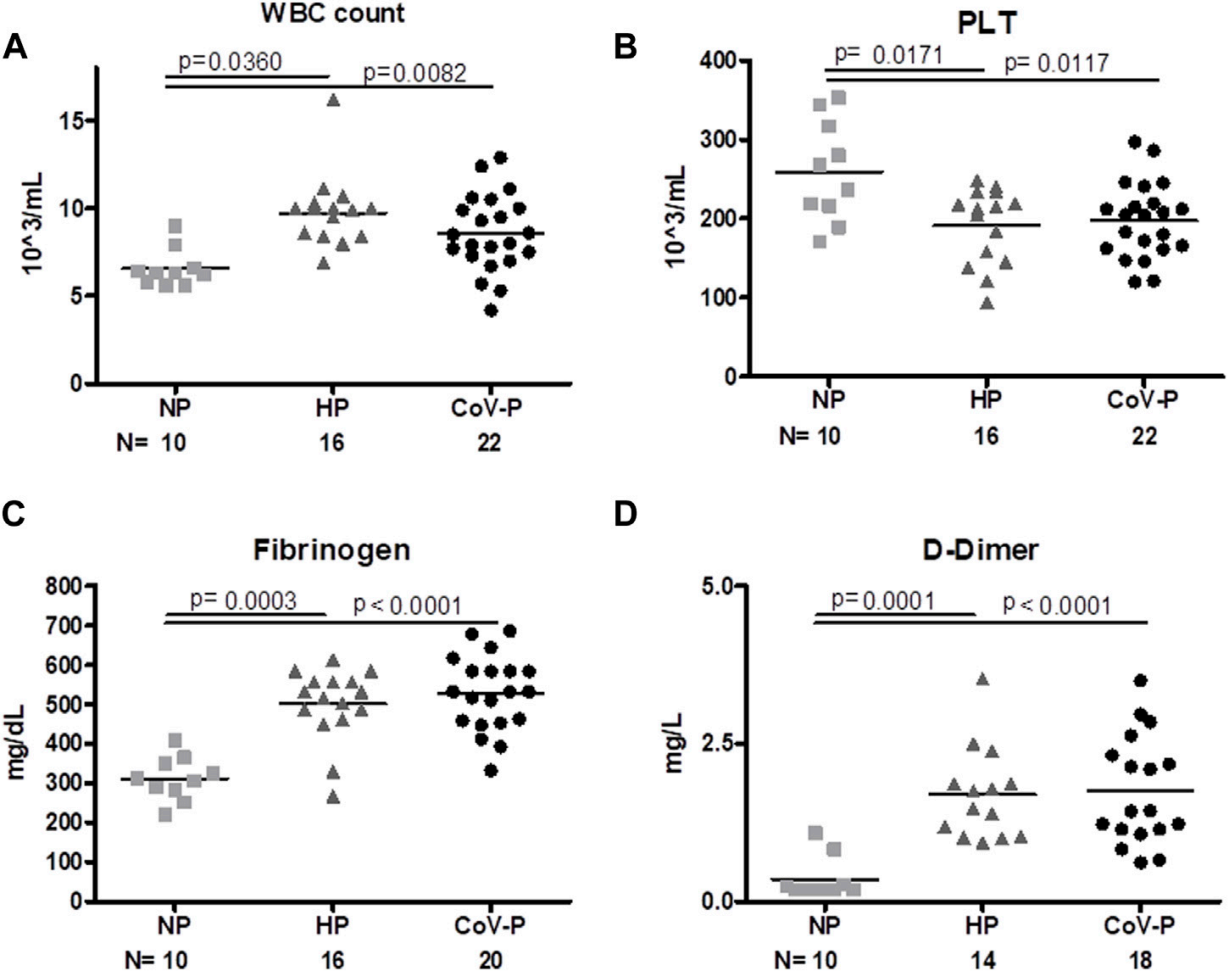
General Laboratory tests. Levels of white blood cells [WBC, 1**(A)**], platelets [PLT, 1**(B)**], fibrinogen [1**(C)**] and D-Dimer [1**(D)**] were measured in healthy non-pregnant subjects (NP), healthy-pregnant (HP), and pregnant subjects infected with SARS-CoV-P (CoV-P). Mann-Whitney *t*-test was preformed to compare the different groups, and *p* < 0.05 was deemed significant. WBC NP vs. HP *p* = 0.0360; NP vs. CoV-P *p* = 0.0082; HP vs. CoV-P *p* = NS. PLT NP vs. HP *p* = 0.0171; NP vs. CoV-P *p* = 0.0117; HP vs. CoV-P *p* = NS. Fibrinogen NP vs. HP *p* = 0.0003; NP vs. CoV-P *p* < 0.0001; HP vs. CoV-P *p* = NS; D-Dimer NP vs. HP *p* = 0.0001; NP vs. CoV-P *p* < 0.0001; HP vs. CoV-P *p* = NS.

Laboratory tests were taken upon enrollment. White Blood Count (WBC), fibrinogen and D-Dimer levels were significantly higher on the day of hospitalization in all pregnant patients regardless of SARS-CoV-2 infection, and levels were within normal range adjusted to pregnancy week. Platelet count was lower in CoV-P and HP compared to NP and within normal range adjusted to pregnancy week (Figure 1; Table 1). A sub-analysis comparing vaccinated CoV-P to non-vaccinated CoV-P subjects did not demonstrate significant differences (Supplementary Table S2).

### 3.4 EVs size, concentration and exosome markers

**FIGURE 2:**
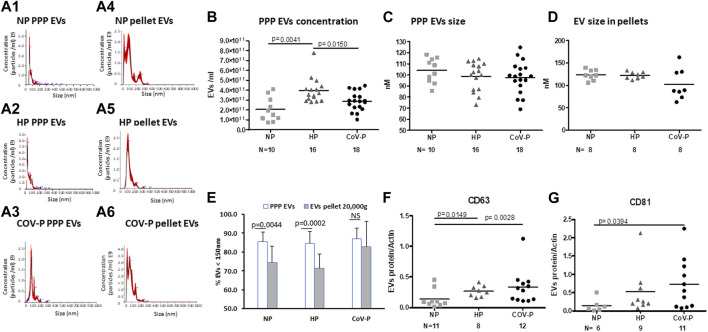
EVs size, concentration and exosome markers. EV size and concentration were analyzed by nanoparticle tracking analysis (NTA) in PPP and in EVs pellet obtained after centrifugation at 20,000 g. Representative graphs of EVs size distribution of PPP and EVs pellets are displayed in Figures 2A1–6. Analysis of PPP EV concentration **(B)** size **(C)** and EV size in pellets **(D)** is presented. Percent of small EVs in PPP and in pellet **(E)** is presented. EV exosome markers CD63 **(F)** and CD81 **(G)** in the study cohort were assessed by western blot and expressed as ratio of actin. Mann-Whitney *t*-test was preformed to compare the different groups, *p* < 0.05 was deemed significant. Concentration NP vs. HP *p* = 0.0041; NP vs. CoV-P *p* = NS; HP vs. CoV-P *p* = 0.0150; Mean EV size and the percentage of small EVs were similar across the three study groups in PPP as well across the three study groups in the pellet. The percentage of small EVs in the pellet of NP and HP was significantly lower than their percentage in PPP (*p* = 0.0044, *p* = 0.0002, respectively), while the percentage of small EVs in pellet and PPP of CoV-P patients was found to be similar. CD63 NP vs. HP *p* = 0.0149; NP vs. CoV-P *p* = 0.0028; HP vs. CoV-P *p* = NS; CD81 NP vs. HP *p* = NS; NP vs. CoV-P *p* = 0.0394; HP vs. CoV-P *p* = NS.

Representative graphs of EVs size distribution of PPP and EV size in pellets obtained by 20,000 g are displayed in Figures 2A1–6. EV concentration was significantly higher in HP (3.945E+11 ± 1.26E+11 EVs/mL) compared to NP women (2.06E+11 ± 1.221E+11EVs/mL), *p* = 0.0041. EV concentration was lower in CoV-P subjects (2.8782E+11 ± 9.7E+10 EVs/mL) compared to HP (3.945E+11 ± 1.26E+11), *p* = 0.0150, (Figure 2B). PPP EVs and EVs pellet include both large and small EVs. The NTA display that EV pellets did not contain EVs in sizes of 500 nm or above (Supplementary Figure S1). Therefore, it can be concluded that EV pellets do not include apoptotic bodies [size of 500 nm–2 μm (Battistelli and Falcieri, 2020)]. The majority of EVs were smaller than 150 nm in both PPP and pellet samples. Mean EV size and the percentage of small EVs were similar across the three study groups in PPP (Figures 2C, E) as well as across the three study groups in the pellet (Figures 2D, E). However, the percentages of small EVs in the pellets of NP and HP were significantly lower than their percentages in PPP (*p* = 0.0044, *p* = 0.0002, respectively), while the percentages of small EVs in pellet and PPP of CoV-P patients were found to be similar.

EVs of CoV-P subjects expressed higher levels of exosome markers CD63 and CD81 compared to NP (*p* = 0.0028, *p* = 0.0394, respectively) (Figures 2F, G). A sub-analysis comparing vaccinated CoV-P to non-vaccinated CoV-P subjects did not demonstrate significant differences in EVs characteristics (Supplementary Table S2). This table presents a comparison of the results of CoV-P subjects in each parameter according to their vaccination status. The table demonstrates that there was no significant difference in the results as related to the status of vaccination. Yet, the comparisons in Supplementary Table S2 may be underpowered to detect significant differences between the groups, due to the small sample size. Expression of the marker Calnexin was found only in cell lysate, indicating that EV pellets were not contaminated with cells (Supplementary Figure S2).

### 3.5 EVs placental marker ACE-2 and TMPRSS2

**FIGURE 3:**
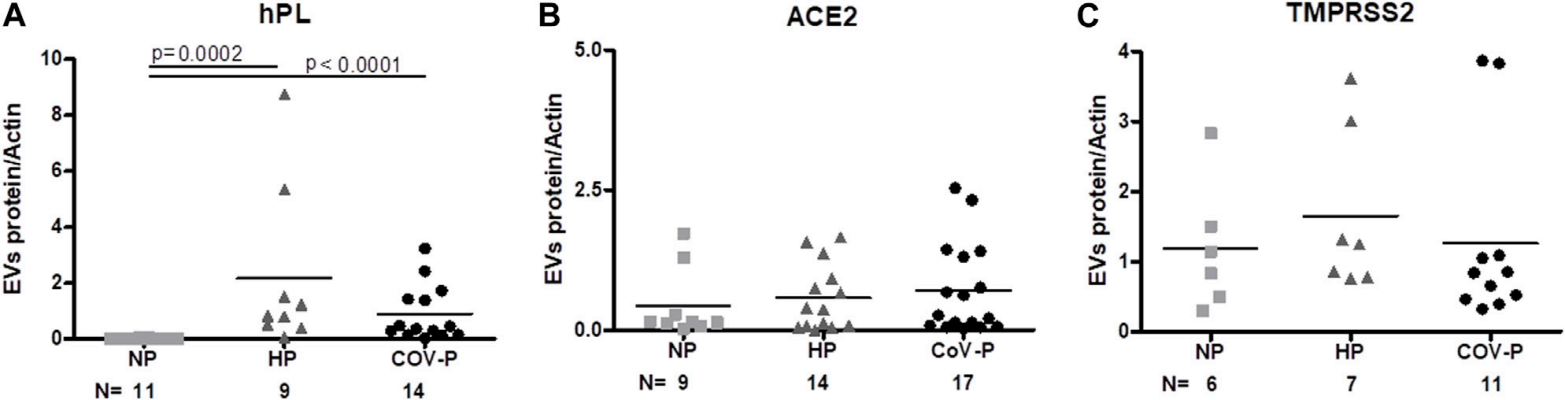
EVs placental marker and SARS-CoV-2 cell entry proteins. EV protein to actin ratio assessed by western blot in the different study groups: NP, HP and CoV-P. EVs’ placental marker hPL **(A)**. EV expression of ACE2 **(B)** and TMPRSS2 **(C)**. Mann-Whitney *t*-test was preformed to compare the different groups, *p* < 0.05 was deemed significant. hPL NP vs. HP *p* = 0.0002; NP vs. CoV-P *p* < 0.0001; HP vs. CoV-P *p* = NS. ACE2 and TMPRSS2 showed no significant difference between all groups.

To validate the placental origin of the EVs, hPL protein levels in EV samples were assessed. As expected, hPL protein levels were not detectable in NP, but were significantly elevated in the EVs of pregnant patients. There was no significant difference in expression of hPL between HP and CoV-P subjects (Figure 3A; Supplementary Figure S2.). Similar levels of ACE-2 and TMPRSS2, proteins that facilitate adhesion and entry of SARS-CoV-2 into recipient cells, were found in the EVs of all three study cohorts (Figures 3B, C; Supplementary Figure S2). Similar levels were also found in EVs of vaccinated and non-vaccinated CoV-P subject EVs (Supplementary Table S2).

### 3.6 EVs cytokine cargo and immune cells markers

**FIGURE 4:**
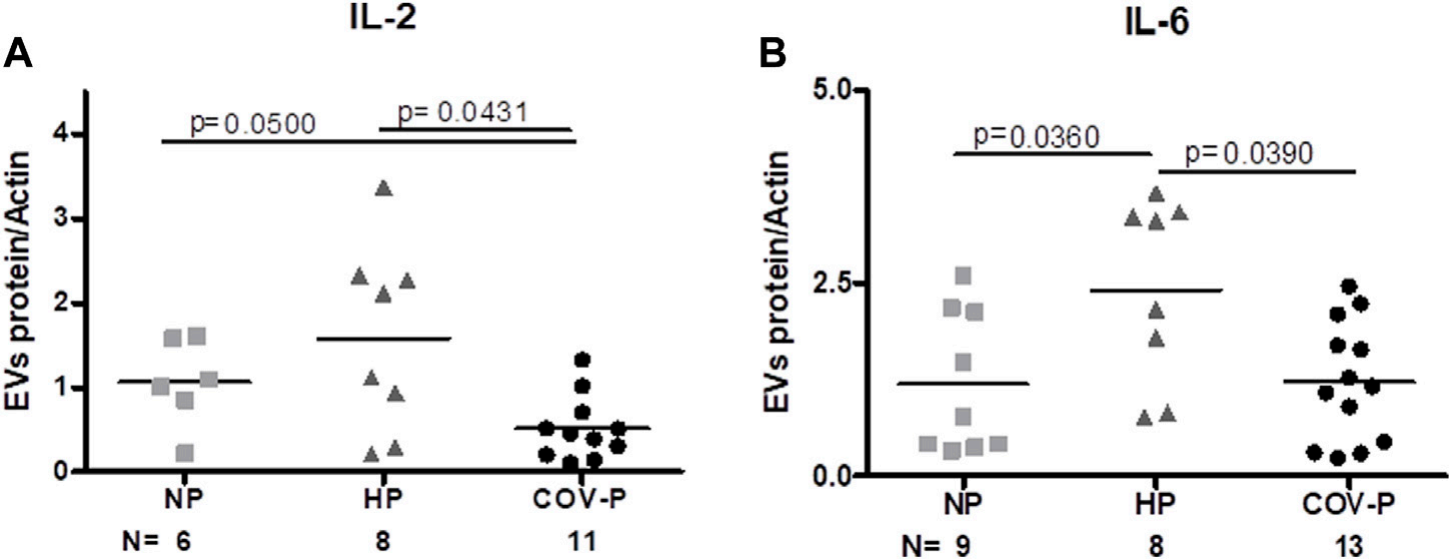
CoV-P EVs display lower levels of IL-2 and IL-6. EVs’ protein to actin ratio assessed by western blot. EV expression of IL-2 **(A)** and IL-6 **(B)** in the different study groups is displayed. Mann-Whitney *t*-test was preformed to compare the different groups, *p* < 0.05 was deemed significant. IL-2 NP vs. HP *p* = NS; NP vs. CoV-P *p* = 0.05; HP vs. CoV-P *p* = 0.0431; IL-6 NP vs. HP *p* = 0.0360; NP vs. CoV-P *p* = NS; HP vs. CoV-P *p* = 0.0390.

IL-2 was significantly lower in CoV-P subjects, compared to NP and HP subjects (*p* = 0.0431) (Figure 4A; Supplementary Figure S2). To assess whether this effect is related to difference in pregnancy week in the study cohort, we performed a sub-analysis of CoV-P subjects at term pregnancy compared to HP (Supplementary Figure S3A). This showed a similar trend with lower levels of IL-2 in CoV-P subjects at 36–41 weeks of gestation (*p* = 0.054). IL-6 concentration was also lower in CoV-P subjects compared to HP (Figure 4B; Supplementary Figure S2) (*p* = 0.0390). Sub-analysis of CoV-P subjects at term pregnancy compared to HP (Supplementary Figure S3B) demonstrated lower levels of IL-6 in CoV-P subjects at term as well (*p* = 0.035). Other cytokines contained within the EVs, such as IL-17, TNF-α, TGF-β and IFN-γ, did not significantly differ between groups (Supplementary Table S3).

**FIGURE 5:**
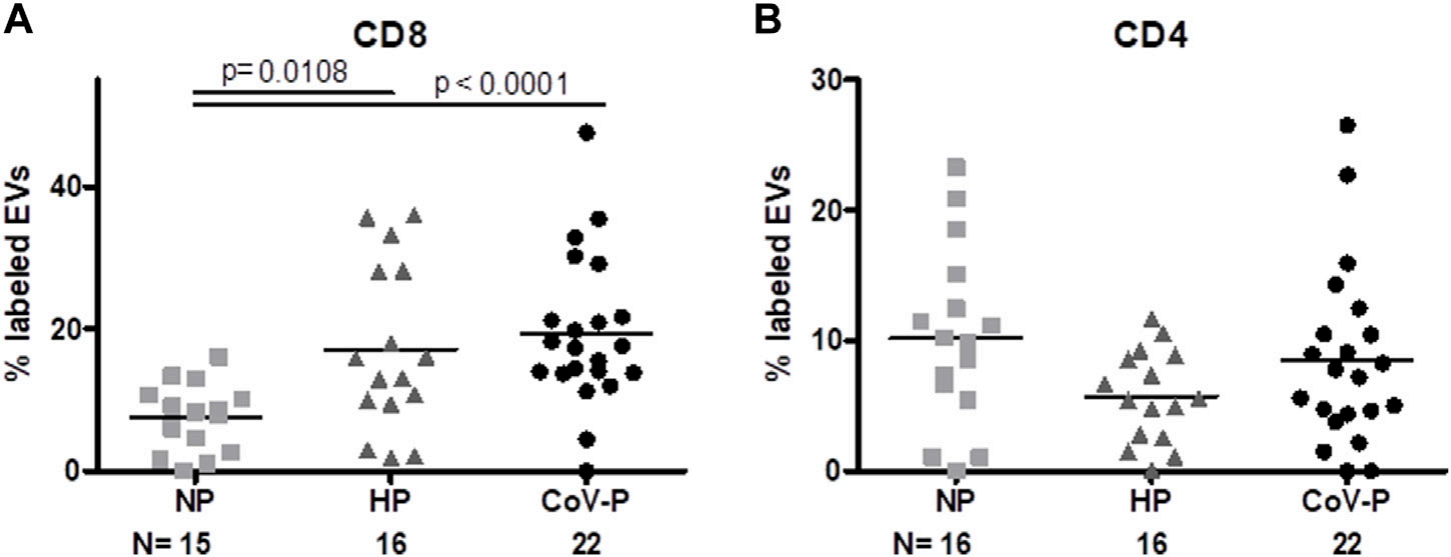
EVs T cell markers expression. EV membrane antigen expression by flow cytometry, expressed as percentage of labeled EVs. EV expression of T cytotoxic CD8 **(A)** and T helper CD4 cell markers **(B)** in NP, HP, CoV-P. Mann-Whitney *t*-test was preformed to compare the different groups, *p* < 0.05 was deemed significant. CD8 NP vs. HP *p* = 0.0108; NP vs. CoV-P *p* < 0.0001; HP vs. CoV-P *p* = NS; CD4 showed no significant difference between all groups.

Expression of T cell markers differed between the study groups. Specifically, EV expression of CD8, a cytotoxic T-cell marker (Figure 5A; Supplementary Figure S4), was higher in both HP and CoV-P compared to NP (*p* = 0.0108, *p* < 0.0001 respectively). The mean expression of T helper cells (Figure 5B; Supplementary Figure S4) (CD4) appeared similar in all three groups. EV expression of other immune cell markers was similar in the study groups. These included FOXP3 (Treg), CD14 (monocyte), CD11a (leucocyte), CD19 and CD22 (B-Lymphocyte), CD28 (T cells, co-stimulatory signals required for T cell activation and survival) and HLA-DR (Table 2). A sub-analysis comparing cytokine cargo and immune cell markers in EVs of vaccinated CoV-P subjects and non-vaccinated CoV-P subjects did not demonstrate significant differences (Supplementary Table S2).

**TABLE 2:** FACS analysis of EV cell markers expressed as percentage of labeled EVs.

Variable	NP	HP	CoV-P	*p*-value NP vs. HP	*p*-value NP vs. CoV-P	*p*-value HP vs. CoV-P	ANOVA
Monocytes (CD14)	1.06 ± 1.1 *n* = 10	2.13 ± 3 *n* = 16	4.71 ± 7.19 *n* = 21	0.2543	0.6766	0.5613	0.9603
Leukocytes (CD11a)	6.31 ± 3.32 *n* = 10	5.38 ± 2.99 *n* = 15	5.07 ± 3.97 *n* = 21	0.7184	0.1733	0.4673	0.4045
B cell receptor (CD22)	9.96 ± 3.57 *n* = 10	12.65 ± 8.35 *n* = 15	13.91 ± 9.42 *n* = 21	0.6926	0.2999	0.6682	0.608
HLA-DR	17.32 ± 10.26 *n* = 10	18.42 ± 11.62 *n* = 16	16.85 ± 9.58 *n* = 21	0.8952	0.9474	0.9366	0.9901
EVs T cytotoxic (CD8)	7.55 ± 4.82 *n* = 15	17.11 ± 11.67 *n* = 16	19.33 ± 10.57 *n* = 21	0.0108	0.0005	0.3672	0.0004
EVs T helper (CD4)	10.2 ± 6.86 *n* = 15	5.73 ± 3.51 *n* = 16	8.45 ± 6.78 *n* = 22	0.0622	0.3366	0.3672	0.1829
EVs T cell co-stimulatory signal (CD28)	5.15 ± 3.71 *n* = 16	6.9 ± 3.5 *n* = 16	5.81 ± 8.5 *n* = 22	0.3965	0.0346	0.0668	0.1484
EVs T reg (FOXP3)	1.37 ± 0.91 *n* = 10	1.9 ± 1.7 *n* = 10	1.34 ± 0.57 *n* = 9	0.7304	0.8148	0.743	0.8842
EVs EC adhesion molecule (CD144)	19.24 ± 16.91 *n* = 15	6.61 ± 9.85 *n* = 16	11.19 ± 11.57 *n* = 22	0.0093	0.1207	0.1207	0.0226
EVs Platelet (CD62p)	0.95 ± 1.54 *n* = 10	0.84 ± 0.98 *n* = 16	1.8 ± 1.67 *n* = 22	0.8533	0.0328	0.0371	0.0364
EVs Endothelial protein C receptor (EPCR)	11.88 ± 4.46 n = 10	10.61 ± 5.64 n = 15	8.80 ± 8.11 n = 21	0.5658	0.024	0.137	0.0632
EVs Thrombomodulin (CD141)	16.74 ± 14.02 *n* = 10	4.71 ± 6.04 *n* = 15	5.77 ± 7.73 *n* = 21	0.0649	0.0401	0.7589	0.0746
EVs Tissue Factor (CD142)	6.17 ± 5.02 *n* = 16	6.27 ± 3.99 *n* = 16	8.57 ± 7.08 *n* = 22	0.6923	0.2312	0.4247	0.4463
EVs TF activity (pM)	139.3 ± 15.27 *n* = 10	144.2 ± 27 *n* = 16	148.1 ± 19.01 *n* = 22	0.9452	0.3568	0.2703	0.6023

Results displayed as mean ± STD, n = number of samples validated in each test. All variables were tested for normality by Shapiro-Wilk normality test and did not show normal distribution.

*p*-value calculated via Mann-Whitney test or Kruskal- Wallis ANOVA.

### 3.7 Vascular and coagulation EVs markers

**FIGURE 6:**
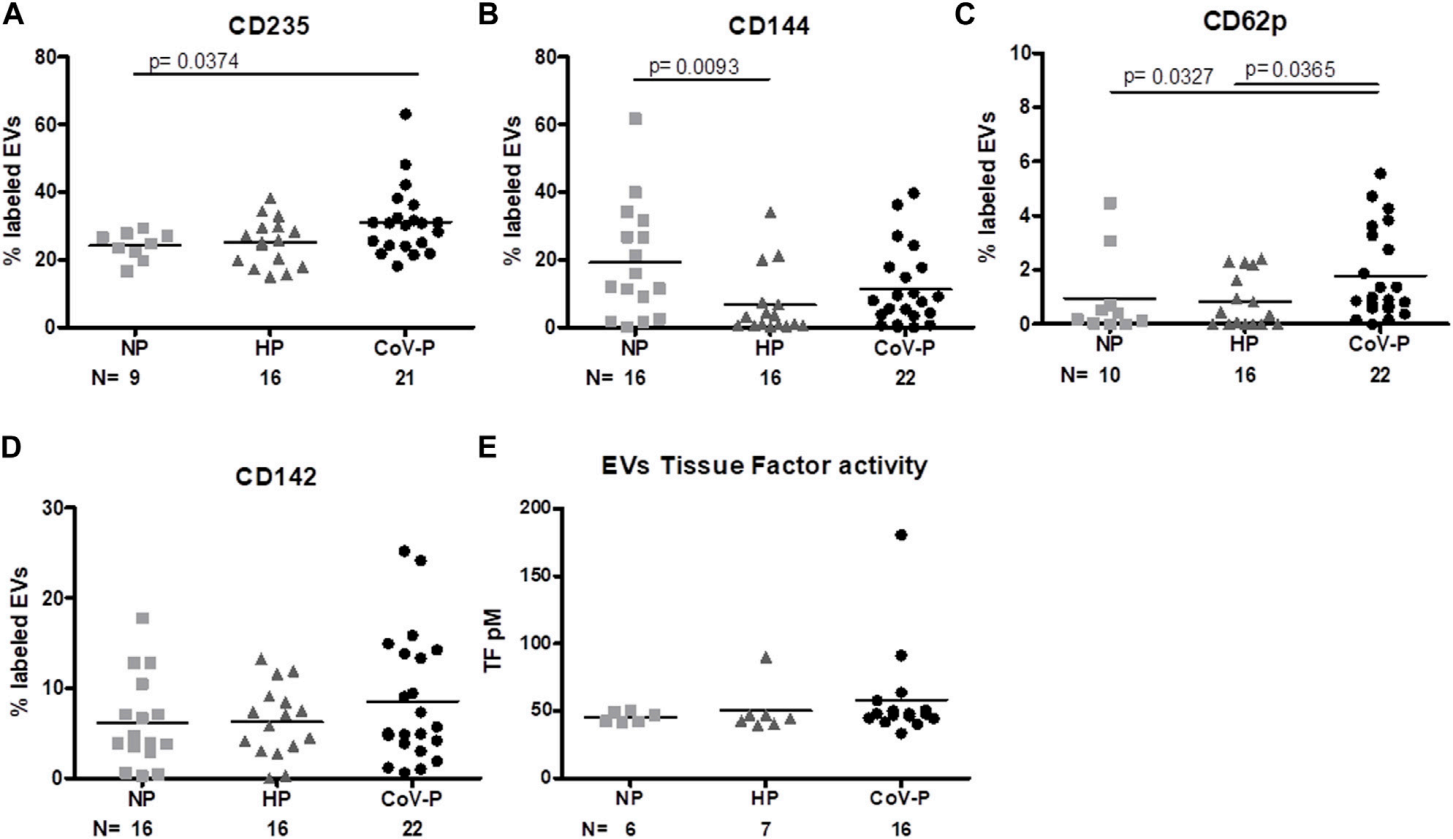
EVs vascular and endothelial marker expression. EV membrane antigen expression by flow cytometry, expressed as a percentage of labeled EVs. NP, HP and CoV-P EVs expression of RBC marker CD235 **(A)**, vascular-endothelial (VE)-cadherin CD144 **(B)**, activated platelet marker CD62P 6 **(C)**, and tissue factor (TF) CD142 **(D)**, are displayed. Levels of TF coagulation activity **(E)** were validated by the Tissue Factor activity assay kit. Mann-Whitney *t*-test was preformed to compare the different groups, *p* < 0.05 was deemed significant. CD235 NP vs. HP *p* = NS; NP vs. CoV-P *p* = 0.0374; HP vs. CoV-P *p* = NS. CD144 NP vs. HP *p* = 0.0093; NP vs. CoV-P *p* = NS; HP vs. CoV-P *p* = NS; CD62p NP vs. HP *p* = NS; NP vs. CoV-P *p* = 0.0327; HP vs. CoV-P *p* = 0.0365; CD142 and tissue factor activity showed no significant difference between all groups.

Expression levels of red blood cell (RBC) marker CD235 were higher in EVs of CoV-P subjects compared to EVs of the NP group (*p* = 0.0374) (Figure 6A; Supplementary Figure S4). Levels of CD144, an EV vascular-endothelial (VE)-cadherin located at junctions between endothelial cells, were significantly lower in HP vs. NP (*p* = 0.0093) (Figure 6B; Supplementary Figure S4). Significantly higher levels of activated platelet marker CD62P (Figure 6C; Supplementary Figure S4) were found in the CoV-P group compared to NP (*p* = 0.0327) or to HP (*p* = 0.0365). Levels of tissue factor (TF) expression (CD142) (Figure 6D; Supplementary Figure S4) and of TF activity (Figure 6E) did not differ between the groups. Other vascular and endothelial markers including endothelial protein C receptor (EPCR), thrombomodulin (CD141), and platelet and endothelial markers (CD62e, CD31 + 41a-, respectively) did not differ between the study groups, nor were there any significant differences in expression of vascular and coagulation markers in vaccinated and non-vaccinated CoV-NP subject EVs (Supplementary Table S2).

## 4 Discussion

While infection with SARS-CoV-2 during pregnancy has been widely studied, and the role of EVs in SARS-CoV-2 in non-pregnant patients has been explored, to the best of our knowledge, the characteristics of EVs in the disease course of pregnant individuals infected with SARS-CoV-2 has not yet been investigated. Clinical manifestations of SARS-CoV-2 vary widely across patients, from asymptomatic disease to severe, acute presentation with multiple organ failure and demise (Long et al., 2022). The corresponding laboratory findings demonstrate severe inflammation and cytokine storm (Merad et al., 2022), with widespread vascular damage (Perico et al., 2021) causing severe mortality. During the recruitment period of our study, we expected to encounter more cases of severe COVID-19 infection. Data from the Centers for Disease Control (CDC) indicates that pregnant individuals are probably more likely to contract SARS-CoV-2 (Lokken et al., 2021) and changes in immunity during pregnancy suggest increased susceptibility of pregnant women to contract SARS-CoV-2 relative to the general population (Jamieson and Rasmussen, 2022). Follow up of the patients revealed that the majority experienced a mild disease without any significant maternal and obstetric complications. Except for three CoV-P subjects, all delivered at term and only two experienced severe disease. Physiological changes, including immunologic and coagulation changes, and decreased lung volume due to uterine growth, are all known to occur during pregnancy. These changes presumably contribute to the increased susceptibility of pregnant women to severe COVID-19 (Wei et al., 2021), and may also explain the clinical complications and rapid deterioration experienced by the severe CoV-P subjects included in our cohort.

The EV profiles of COVID-19 patients are known to correlate directly with disease severity (Krishnamachary et al., 2021) (Balbi et al., 2021). EVs of COVID-19 patients express a variety of cytokines and coagulation factors (Guervilly et al., 2021). Our study found significantly lower EV concentrations in CoV-P subjects compared to the HP group. Previous work has shown that concentrations of EVs in pregnancy are significantly higher compared to non-pregnant individuals (Salomon et al., 2014). Our work reinforced these findings, as EV concentrations were significantly higher in HP than NP. However, EV concentration was significantly lower in CoV-P subjects compared to the HP group, suggesting that COVID-19 infection decreases the concentration of EVs in maternal circulation, or alternatively, it may relate to the earlier gestational age at sampling of COVID-19 patients (Salomon et al., 2016). This reduction was accompanied by a distinct rise in exosomes markers CD63 and CD81 in CoV-P subjects, compared to NP. CD63 marker indicates the endosomal pathway related to part of the small extracellular vesicle formation (Andreu and Yanez-Mo, 2014), and their abundance in CoV-P subjects may reflect alterations to this pathway during SARS-CoV-2 infection. This difference in origin cannot be attributed to a placental source, as we did not see differential expression of placental lactogen in EVs between HP and CoV-P subjects. Previous studies showed that SARS‐CoV‐2, like other viruses (Zika, HIV) interacts with Rab proteins to facilitate exosome release (Babaei et al., 2022), and may be related to the moderate shift to exosome biogenesis that we found in CoV-P subjects. Our study revealed an attenuated inflammatory response in CoV-P subjects. CoV-P EVs expressed a reduction in EV cytokine content compared to other groups. Previous work on cytokine expression in COVID-19 patients revealed a correlation between disease severity and certain cytokine levels (Mahat et al., 2021; Merad et al., 2022). However, our results did not produce similar evidence. Levels of IL-6 and IL-2 were reduced in CoV-P subjects compared to HP. These cytokines play a key role in pregnancy at all stages, from implantation to parturition. IL-6 increases during healthy pregnancy, and both decreased and elevated levels of this cytokine relative to normal are associated with pregnancy complications, including infertility, miscarriage, preterm birth, and preeclampsia (Prins et al., 2012). IL-2 directs T lymphocyte differentiation into effector and memory T cells as well as regulatory T cells which are important for preventing autoimmunity (Darmochwal-Kolarz et al., 2017; Spence et al., 2021). Cytokines direct biological processes throughout pregnancy (Yockey and Iwasaki, 2018), and mediate the balance between inflammation and immune regulation during pregnancy. Although we expected our results to indicate evidence of cytokine storm, our data shows a clear reduction in cytokine expression. Moreover, pro-inflammatory cytokines increase EV expression of ACE2, a part of the soluble form of ACE2 (Scialo et al., 2020). In the current study, EVs of all three study groups showed similar ACE2 expression. A decreased inflammatory cytokine response in CoV-P compared to healthy-pregnant subjects may explain why there is no increase in EV expression of ACE2 in these patients.

Levels of plasma WBCs were also similar between HP and CoV-P and showed only the elevated count expected during pregnancy (Chandra et al., 2012) compared to NP. We did not find significant activation of adaptive immunity between groups. EVs’ cytotoxic T cell (CD8) markers were higher in COV-P and HP compared to NP but there was not a significant increase in CoV-P EVs’ expression of CD8 compared to HP EVs. T Helper cell (CD4) expression was similar between the study cohort EVs.

EVs of CoV-P subjects display evidence of increased expression of the RBC marker (CD235) compared to NP EVs. SARS-CoV-2 attaches to RBCs to induce formation of RBC clumping to endothelial cells (Scheim, 2022). Endothelial marker VE-Cadherin (CD144) was lower in HP compared to NP, reflecting the vascular protective effects that characterize healthy term pregnancies (Groten et al., 2010). This reduction in CD144 was absent in EVs of CoV-P and their mean was higher than HP. VE-Cadherin, the endothelial gap junction protein, maintains the endothelial barrier. In inflammatory states, VE-cadherin undergoes phosphorylation, destabilization, and internalization that directly damages the microvascular endothelial barrier (Xiong et al., 2020). The trend of increased VE-Cadherin expression in EVs detected in the CoV-P group compared to HP EVs did not exceed that of NP EVs, perhaps indicating only slight endothelial damage (Sansone et al., 2018; Bar-Sela et al., 2020). Therefore, we cannot derive any conclusions regarding CoV-P EVs’ coagulability. In our study, we saw minor changes in the coagulation system markers. COVID-19 patients display elevated levels of D-dimer and fibrinogen beginning early in the disease and up to three-fold increases in D-dimer levels. These levels correlate with an overall poor prognosis (Aharon A. et al., 2023). D-dimer and fibrinogen levels are also elevated during pregnancy and increase as the pregnancy progresses (Xie et al., 2014; Abbaszade Dibavar et al., 2018). These changes indicate a significant modification of the fibrinolytic system during pregnancy. In the current study, we did not find any additive effects in the combination of pregnancy and COVID-19 disease in the plasma levels of D-dimer and fibrinogen, as they were found to be similar in HP and COV-P groups. This may be related to the very high levels that characterize pregnancy or it may indicate that the infection of pregnant women with SARS-CoV-2 is controlled and moderate, and therefore no additive effects are observed in these parameters. The only parameter that may have indicated increased coagulation in the CoV-P EVs was activated platelet marker CD62p, which was significantly increased in CoV-P EVs. This elevated activation of platelets did not correspond to plasma platelet counts, as results were found to be similar in HP and COV-P groups. Aside from their role in thrombosis, platelets are key mediators of the inflammatory response (Gros et al., 2014). The membrane marker P-selectin (CD62p) is a surface marker that is upregulated in activation of platelets and though it is suggested to correlate with disease severity (Uzun et al., 2022), other studies have found increased platelet activation in non-severe patients as well (Zaid et al., 2020). However, we must exercise caution when interpreting these results. While we witnessed elevated CD62p in pregnant COVID-19 subjects, the absolute levels of this marker were low overall, and did not exceed 10% expression in all subjects.

In contrast to previous studies, which reported a clear elevation in coagulability (Jacob et al., 2020; Kichloo et al., 2020), we found that EV levels of tissue factor expression (CD142) and activation (TF activity) were not significantly increased in CoV-P subjects.

Conducting a clinical study in pregnant women during the COVID-19 pandemic was challenging. Furthermore, EVs yield is usually low and varies. Therefore analyzing all markers on each sample was practically impossible. The main limitation of our study lies in the small size of the cohort, which prevents us from drawing conclusions from cases without statistically significant differences between groups, due to lack of power in these instances. In this case, the absence of a significant difference between vaccinated and non-vaccinated CoV-P subjects, may be attributed to the small sample size. The limited volumes of the samples acquired from the subjects resulted in different sample sizes in the experiments. The sample size for each experiment appears in Tables 1, 2 and in the Supplementary Table S4. To clarify the effect (or lack of effect) of the number of samples in each experiment, the aggregate characteristics listed in Table 1 (Age, BMI, Vaccinated, Gravidity, Parity, Gestational week, Systolic and Diastolic blood pressure, Heart rate, WBC, PLT, Fibrinogen and D-Dimer) were applied to each of the experiments and presented in the Supplementary Table S5. There are no significant changes between Table 1 and aggregate characteristics in each experiment as presented in Supplementary Table S5. Another limitation was our inability to stratify the CoV-P group according to disease severity due to limited admission of only a few pregnant patients with severe COVID-19 to our hospital during the recruitment period. This was an unexpected limitation of this study, as it was conducted in a tertiary center where we would have expected to encounter more cases of pregnant COVID-19 subjects with all degrees of disease severity during the enrollment period. Another limitation was the difference in distribution of gestational age between CoV-P and HP subjects. While this was an inherent limitation of the study design, it may have affected our results. We assume this has only a minor effect, as we have analyzed our results according to pregnancy age. A Supplementary Table S6 of Spearman correlation clarifies that the results do not correlate to gestational age. The test was performed on all pregnant subjects, HP and CoV-P. Combining the two groups together has allowed us an adequate sample size to perform this test (more than 20 subjects in each comparison (Kelley et al., 2019)). There was no correlation between gestational age and result in any parameter.

Also, unfortunately, we were not able to compare our results to EVs of non-pregnant female SARS-CoV-2-positive subjects because the population of female COVID-19 patients admitted to our hospital during enrollment was much older and had severe co-morbidities, which did not allow them to serve as an appropriate study group for comparison. However, in a parallel study we conducted recently, we found that EVs of COVID-19 patients reflect inflammation, thrombogenicity, and disease severity (Aharon A. et al., 2023). Finally, despite the concept that EV isolation need to be done by high-speed centrifugation (100,000 g), the current study NTA results support the conclusion that EV pellets (isolated by 20,000 g) contained large and small EVs but not apoptotic bodies. Therefore, 100,000 g centrifugation, which isolates mainly small EVs, was not needed for the purpose of this study.

In summary, previous studies suggested that the majority of pregnant individuals experience mild or asymptomatic disease and are not at increased risk for adverse maternal or obstetric outcomes (Caillon et al., 2022) while others reported that pregnant women are at higher risk of severe disease complications and adverse pregnancy outcomes (Saari et al., 2015). This discrepancy led us to inquire whether EVs of pregnant subjects during SARS-CoV-2 infection would reflect disease severity as seen in the non-pregnant population, or whether they would shed light on the positive disease course that most pregnant subjects experience. Progression of a healthy pregnancy requires a delicate balance between immune system activity, cytokine signals and coagulation pathways. EVs reflect and affect this balance and connect between these three important systems. The immune system must protect the fetus and mother from foreign pathogens, while simultaneously attenuating its response, as the fetus is semi-allogeneic and is vulnerable to immune-mediated damage (Yockey and Iwasaki, 2018). This immune tolerance has paradoxical implications during viral infection. While it may leave the mother vulnerable to certain infections, the attenuated response may also reduce the risk of an exaggerated hyper-immunity state. This delicate balance may contribute to the mechanisms underlying the progression of COVID-19 disease in pregnant women.

## 5 Conclusion

While we expected the EVs of pregnant subjects infected with SARS-CoV-2 to reflect a heightened inflammatory and coagulation response, we instead discovered an attenuated inflammatory response. This finding may partially explain the observations reported in previously published work that while pregnant individuals are at higher risk of contracting SARS-CoV-2, most of them experience a mild disease without significant complications.

## Data Availability

The original contributions presented in the study are included in the article/Supplementary Material, further inquiries can be directed to the corresponding author.

## Supplementary material

SUPPLEMENTARY FIGURE S1 EV size in pellets 10th–90th percentile EV size in pellets was analyzed by nanoparticle tracking analysis (NTA) EVs pellet obtained after centrifugation at 20,000 g. The graph presents the 10-90 percentile in the study cohorts.

SUPPLEMENTARY FIGURE S2 Representation of EV protein content by western blot EV pellets obtained from similar PPP volumes (250 ul) were lysed. Samples of NP (healthy non—pregnant), HP (healthy-pregnant) subjects, and CoV-P- pregnant subjects infected with SARS-CoV-P were loaded and separated on 4-20% Precast Protein Gels and then transferred to nitrocellulose membranes. Membranes were washed and immunoblotted. The membranes were incubated with specific antibodies.

SUPPLEMENTARY FIGURE S3 Sub analysis of cytokine content in term pregnancies Levels of EV cytokine content from subjects that were in their third trimester of pregnancy, from 37 to 41 weeks of gestation, were compared between HP- healthy-pregnant subjects and CoV-P pregnant subjects infected with SARS-CoV-P. Levels of IL-2 **(A)** and IL-6 **(B)** are compared between the two groups. IL-2 HP vs CoV-P at term showed a non-significant trend of decrement. IL-6 expression was significantly decreased in CoV-P at term compared to HP, *p*= 0.0350.

SUPPLEMENTARY FIGURE S4 Representations of EV membrane antigen expression by flow cytometry EV gates were set using Megamix, a mix of fluorescent beads (beads size 0.3/0.9/3 µm) S4 **(A)** and 0.2 µm polystyrene beads (Malvern Pananalytical, United Kingdom) S4 **(B)**. EV membrane antigen levels were assessed by flow cytometry using fluorescent antibodies. Events were collected by time at a flow rate of 10 µL per minute. Controls and samples were analyzed in the same acquisition setting and reagent conditions. Instrument configuration and settings: Gain: FSC 500; SSC 100; Violet SSC 40; PE 120; APC400; FITC 100, Threshold: manual 10000 height. PBS only is presented (4c1). Unstained controls (S4c2-4). Isotype controls of PBS, NP, HP and CoV-P are presented in 4d-f. Stained samples of PBS, NP, HP and CoV-P are presented in S4g-l.
