# Supplementary material for: Characterization of extracellular vesicles in COVID-19 infection during pregnancy
Source: Front Cell Dev Biol. 2023 Jul 25;11:1135821. doi: 10.3389/fcell.2023.1135821 (PMC10407400; doi:10.3389/fcell.2023.1135821)
Supplement: Supplementary file 1 [file DataSheet2.pdf]

Supplementary table S5.

Figure 2

NTA	NP=10	HP=16	CoV-P=18	p value NP vs HP	p value NP vs CoV-P	p value HP vs CoV-P	ANOVA
Age (years)	35 [30.5, 38.5]	33 [31, 36.5]	32 [29, 36]	0.5797	0.3124	0.5399	0.5472
BMI	23.57±2.72	23.84±2.99	23.32±4.02	0.937	0.6732	0.6376	0.854
Vaccinated	10 (100%)	16 (100%)	6 (33.33%)	1	**<0.0001**	**<0.0001**	**<0.0001**
Gravidity (number pregnancies)	1.5 [2, 3]	2 [1, 2.5]	3 [1, 5]	0.4265	0.0605	0.2051	0.1211
Parity (number of deliveries)	1.5 [0, 2]	1 [0, 1.5]	2 [0, 3]	0.5059	0.3312	0.0514	0.1196
Gestational week	N/A	39 [39, 40]	36 [29, 38]	N/A	N/A	**0.0003**	N/A
Systolic blood pressure (mmHg)	117.3±9.63	115.6±6.41	118.1±11.46	0.7555	0.7305	0.3045	0.6
Diastolic blood pressure (mmHg)	73.11±7.96	67.88±7.93	69.21±8.53	0.1929	0.3499	0.631	0.403
Heart rate (bpm)	76±9.21	82.75±9.75	89.84±16.21	0.0894	**0.0171**	0.1157	**0.0249**
WBC (10^3/mL)	6.55±1.09	9.273±1.16	8.216±2.11	**0.0002**	**0.0218**	0.0564	**0.0007**
PLT (10^3/mL)	260.1±62.65	198.3±46.68	196.5±49.29	**0.0307**	**0.0116**	0.5734	**0.0261**
Fibrinogen (mg/dL)	311.4±54.57	501.3±91.88	530.6±98.09	**0.0003**	**<0.0001**	0.3969	**<0.0001**
D-Dimer (mg/L)	0.356±0.32	1.695±0.73	1.596±0.77	**0.0001**	**0.0002**	0.7767	**<0.0001**

Figure 2

CD63	NP=11	HP=8	CoV-P=12	p value NP vs HP	p value NP vs CoV-P	p value HP vs CoV-P	ANOVA
Age (years)	35.27±7.32	32.33±5.05	33.5±6.9	0.2231	0.5173	0.6184	0.4665
BMI	24.36±3.62	23.11±2.81	23.55±4.01	0.4826	0.6891	0.7285	0.7475
Vaccinated	11 (100%)	8 (100%)	3 (25%)	1	**0.0001**	**0.0001**	**0.0001**
Gravidity (number pregnancies)	2 [0,5]	3 [2, 4.5]	4 [1.5,8.5]	0.4643	0.0817	0.3128	0.1848
Parity (number of deliveries)	2 [0,3]	1 [0.5, 2]	2 [0.5, 4.25]	0.6373	0.5847	0.1252	0.3761
Gestational week	N/A	39 [38.5, 40]	36.5 [34, 37]	N/A	N/A	**0.0373**	N/A
Systolic blood pressure (mmHg)	121.3±12.67	114.8±9.67	121.1±12.39	0.2864	0.666	0.1542	0.3091

Diastolic blood pressure (mmHg)	75.18±6.31	67.75±9.35	67.42±9.58	0.0982	0.0788	0.9077	0.1223
Heart rate (bpm)	76.91±8.68	83.22±12.3	91.5±17.78	0.2692	**0.0245**	0.2261	0.0599
WBC (10^3/mL)	6.6±1.14	9.2±1.03	8.508±2.21	**0.0017**	**0.0156**	0.1997	**0.0029**
PLT (10^3/mL)	267.2±62.27	182.4±56.05	196.8±37.71	**0.0188**	**0.0085**	0.7222	**0.0146**
Fibrinogen (mg/dL)	321.3±47.31	478.6±107.4	500.7±97.86	**0.0078**	**0.0006**	0.9091	**0.0018**
D-Dimer (mg/L)	0.3744±0.34	1.595±0.47	2.095±0.87	**0.0011**	**0.0004**	0.2004	**0.0002**

Figure 2

**CD81**	NP=6	HP=9	CoV-P=11	p value NP vs HP	p value NP vs CoV-P	p value HP vs CoV-P	ANOVA
Age (years)	36.5 [33.25,38.5]	33 [29, 38.5]	31 [27, 38]	0.4081	0.3648	0.7608	0.5628
BMI	23.74±3.15	23.39±2.51	24.05±4.52	0.6797	0.9599	0.5949	0.8252
Vaccinated	6 (100%)	9 (100%)	2 (18.18%)	1	**< 0.0001**	**< 0.0001**	**< 0.0001**
Gravidity (number pregnancies)	2.5 [0.75, 4.25]	3 [1, 4.4]	3 [1, 7]	0.811	0.2841	0.3302	0.4298
Parity (number of deliveries)	2 [0,3]	1 [0,2]	2 [0,3]	0.2379	0.9584	0.1523	0.2748
Gestational week	N/A	39 [39, 39.5]	37 [34, 40]	N/A	N/A	0.0631	N/A
Systolic blood pressure (mmHg)	118.8±7.52	117.9±8.13	116.9±15.54	0.8593	0.9626	0.8868	0.973
Diastolic blood pressure (mmHg)	74.83±6.21	68.22±8.86	69.55±10.75	0.1749	0.4504	0.7598	0.4306
Heart rate (bpm)	75.83±4.92	83±11.34	96±18.18	0.262	**0.0089**	0.102	**0.0208**
WBC (10^3/mL)	6.833±1.36	8.956±1.17	9.309±2.08	**0.0132**	**0.0181**	0.9696	**0.0212**
PLT (10^3/mL)	288.2±60.35	189.8±54.79	190.2±39.65	**0.0176**	**0.0042**	0.8196	**0.0111**
Fibrinogen (mg/dL)	335.3±53.14	460.4±97.35	514.8±102.9	**0.0291**	**0.0042**	0.2701	**0.0082**
D-Dimer (mg/L)	0.2117±0.03	1.674±0.57	1.955±0.84	**0.0022**	**0.001**	0.5357	**0.0012**

Figure 3

**hPL**	**NP=11**	**HP=9**	**CoV-P=14**	**p value NP vs HP**	**p value NP vs CoV-P**	**p value HP vs CoV-P**	**ANOVA**
Age (years)	37 [29.75, 39.5]	30.5 [27.25, 33]	31 [27, 38]	0.0653	0.3845	0.4814	0.2084
BMI	25±3.93	23.48±2.74	23.82±4.22	0.4515	0.5634	0.7396	0.6742
Vaccinated	11(100%)	9 (100%)	4 (28%)	1	**< 0.0001**	**< 0.0001**	**< 0.0001**
Gravidity (number pregnancies)	3 [0, 5]	2 [1.25, 3.75]	3 [1, 5]	0.9612	0.4172	0.3319	0.5466

Parity (number of deliveries)	2 [0,4]	2 [1.25, 2.75]	3 [1, 3]	0.8823	0.3135	0.3663	0.4925
Gestational week	N/A	39 [39, 40]	35 [33, 37.75]	N/A	N/A	**0.0113**	N/A
Systolic blood pressure (mmHg)	120.4±13.69	113.1±8.58	119±14.69	0.2884	0.849	0.2141	0.3806
Diastolic blood pressure (mmHg)	75.11±5.62	65.63±9.47	69.36±10.49	**0.048**	0.2695	0.4297	0.1382
Heart rate (bpm)	75±7.0	86.13±11.23	92.09±19.59	**0.048**	**0.0274**	0.4822	**0.0395**
WBC (10^3/mL)	6.55±1.27	8.975	8.627	**0.0116**	**0.0393**	0.3633	**0.0221**
PLT (10^3/mL)	257.8±66.66	192±55.96	190.7±44.44	**0.1812**	**0.0394**	0.9671	**0.1243**
Fibrinogen (mg/dL)	318.1±39.53	475.6±114.4	529±88.5	**0.0293**	**0.0014**	0.3967	**0.0047**
D-Dimer (mg/L)	0.36±0.35	1.437±0.39	2.07±0.83	**0.0124**	**0.0017**	0.1404	**0.0017**

Figure 3

**ACE2**	**NP=9**	**HP=14**	**CoV-P=17**	**p value NP vs HP**	**p value NP vs CoV-P**	**p value HP vs CoV-P**	**ANOVA**
Age (years)	37 [30.5, 39]	33 [30.5, 38.5]	34 [28, 37.5]	0.4608	0.2684	0.9165	0.5459
BMI	24.42±3.58	23.11±2.62	24.14±3.77	0.3164	0.9323	0.3238	0.491
Vaccinated	9 (100%)	14 (100%)	5 (29.4%)	1	**< 0.0001**	**< 0.0001**	**< 0.0001**
Gravidity (number pregnancies)	2 [0, 4.5]	2 [1,3.5]	3 [1, 6]	0.6355	0.1404	0.2263	0.2439
Parity (number of deliveries)	2 [0,3]	1 [1, 2.5]	2 [0,2.5]	0.7306	0.9102	0.8295	0.9336
Gestational week	N/A	39 [39,40]	36 [28.5,37]	N/A	N/A	**0.0014**	N/A
Systolic blood pressure (mmHg)	121.2±13.54	117.4±7.10	117.5±13.28	0.7632	0.914	0.6752	0.8958
Diastolic blood pressure (mmHg)	72.78±6.7	69.08±7.72	68.12±9.62	0.3325	0.2921	0.8174	0.495
Heart rate (bpm)	77.67±8.82	79.92±10.67	94.47±18.85	0.6637	**0.0177**	**0.0169**	**0.0143**
WBC (10^3/mL)	6.313±0.75	9.754±2.27	8.388±2.37	**0.0003**	**0.0183**	0.1021	**0.0011**
PLT (10^3/mL)	263.3±60.09	190.2±48.19	197.2±43.32	**0.0125**	**0.0122**	0.867	**0.0189**
Fibrinogen (mg/dL)	305.3±58.01	486.3±95.36	527.3±99.68	**0.0016**	**0.0003**	0.3325	**0.0004**
D-Dimer (mg/L)	0.28±0.22	1.528±0.54	1.831±0.88	**0.0002**	**0.0002**	0.4207	**0.0001**

Figure 3

**TMPRSS2**	**NP=6**	**HP=7**	**CoV-P=11**	**p value NP vs HP**	**p value NP vs CoV-P**	**p value HP vs CoV-P**	**ANOVA**

Age (years)	36.5 [30.5, 395]	33 [31, 39]	32 [29,38]	0.7308	0.4505	0.7511	0.7143
BMI	23.12±2.62	24.2±2.75	24.59±4.47	0.8357	0.2635	0.6691	0.5721
Vaccinated	6 (100%)	7 (100%)	3 (27.27%)	1	**0.0008**	**0.0008**	**0.0008**
Gravidity (number pregnancies)	2.5 [0,4.5]	2 [2,3]	5 [1, 9]	1	0.2474	0.6174	0.5486
Parity (number of deliveries)	2 [0, 3]	2 [1, 3]	3 [1, 6]	0.9452	0.2906	0.4366	0.4787
Gestational week	N/A	40 [39, 40]	36 [29, 37]	N/A	N/A	**0.0185**	N/A
Systolic blood pressure (mmHg)	117.7±7.37	118.3±7.48	118.5±14.34	0.8357	0.5796	0.7512	0.8228
Diastolic blood pressure (mmHg)	72±7.27	66.71±8.48	67.82±10.51	0.366	0.5136	0.9638	0.6362
Heart rate (bpm)	77.83±9.91	79.57±13.84	90.36±11.6	1	0.0501	0.0772	0.0671
WBC (10^3/mL)	6.517±0.76	9.871±3.11	8.027±1.8	**0.0023**	**0.0308**	0.2049	**0.0103**
PLT (10^3/mL)	270.3±67.4	200.4±50.61	206.9±48.58	**0.1375**	**0.1078**	0.8562	**0.1825**
Fibrinogen (mg/dL)	306.9±68.2	488.7±112.4	489.3±105.1	**0.014**	**0.0028**	0.7577	**0.0101**
D-Dimer (mg/L)	0.2033±0.03	1.36±0.35	1.876±0.94	**0.0022**	**0.0004**	0.3884	**0.0016**

Figure 4

**IL-2**	**NP=6**	**HP=8**	**CoV-P=11**	**p value NP vs HP**	**p value NP vs CoV-P**	**p value HP vs CoV-P**	**ANOVA**
Age (years)	37 [32.55,41.25]	32 [28.5, 34.5]	31 [27,39]	0.0915	0.1906	0.967	0.2249
BMI	24.12±3.21	23.57±2.69	22.95±3.65	0.6166	0.3385	0.8563	0.6233
Vaccinated	6 (100%)	8 (100%)	3 (27%)	1	**0.0001**	**0.0001**	**0.0001**
Gravidity (number pregnancies)	2 [0, 4.25]	2.5 [2,3.75]	3 [1,5]	0.6011	0.3043	0.7678	0.5823
Parity (number of deliveries)	1 [0,3.5]	1 [0.25, 1.75]	2 [0,2]	0.8405	1	0.5721	0.8593
Gestational week		39 [39,40]	34 [28,37]	N/A	N/A	**0.0125**	N/A
Systolic blood pressure (mmHg)	117.8±9.64	114.9±10.66	117.5±13.79	0.6503	0.9598	0.5621	0.4504
Diastolic blood pressure (mmHg)	74.33±6.02	67.38±9.58	67.91±8.85	0.2185	0.2068	0.9338	0.3374
Heart rate (bpm)	73±4.86	82±11.46	94.64±18.4	0.1619	**0.005**	0.1374	**0.0131**
WBC (10^3/mL)	6.66±1.37	8.975	9.1	**0.0231**	**0.0414**	0.967	**0.0492**
PLT (10^3/mL)	285±48.94	184.1±61.41	200.3±37.21	**0.0186**	**0.0065**	0.5913	**0.0147**

Fibrinogen (mg/dL)	308.5±41.72	470.2±112.4	536.2±100.3	**0.0335**	**0.0022**	0.3849	**0.0068**
D-Dimer (mg/L)	0.344±0.27	1.481±0.37	2.077±0.84	**0.0057**	**0.0022**	0.1739	**0.0019**

Figure 4

**IL-6**	**NP=9**	**HP=8**	**CoV-P=13**	**p value NP vs HP**	**p value NP vs CoV-P**	**p value HP vs CoV-P**	**ANOVA**
Age (years)	37 [31.5, 39]	32 [27.25, 34.5]	31 [27.5,37.5]	0.1225	0.2696	0.7996	0.2917
BMI	23.56±2.89	23.35±2.56	24.25±4.1	0.8098	0.8412	0.447	0.769
Vaccinated	9 (100%)	8 (100%)	3 (27%)	1	**0.0001**	**0.0001**	**0.0001**
Gravidity (number pregnancies)	2 [0,4.5]	2 [1.25, 3]	3 [2,6]	0.392	0.5115	0.1197	0.1383
Parity (number of deliveries)	2 [0,3]	1 [0,1]	2 [0.5, 2.5]	0.8405	0.6759	0.0506	0.187
Gestational week	N/A	39 [39, 40]	36 [34,37]	N/A	N/A	**0.0403**	N/A
Systolic blood pressure (mmHg)	117±8.08	114±10.07	117.7±15	0.5307	0.6161	0.4463	0.6427
Diastolic blood pressure (mmHg)	74.33±6.245	67.25±9.68	68.46±10.72	0.1622	0.2845	0.8271	0.341
Heart rate (bpm)	73.89±5.71	82.25±11.88	93.54±18.41	0.1368	**0.0089**	0.0822	**0.0122**
WBC (10^3/mL)	6.625±1.22	9.100±1.06	8.808±2.38	**0.0045**	**0.0168**	0.4463	**0.0085**
PLT (10^3/mL)	271±65.45	178.0±58.2	194.1±37.45	**0.0207**	**0.0073**	0.5621	**0.0135**
Fibrinogen (mg/dL)	325.9±48.38	503.4±77.19	523.4±105.18	**0.0027**	**0.0006**	0.7716	**0.0008**
D-Dimer (mg/L)	0.3975±0.35	1.481±0.37	2.039±0.85	**0.0057**	**0.0022**	0.1739	**0.0004**

Figure 5/6

**FACS (CD4 CD8 CD142 CD144)**	**NP=16**	**HP=16**	**CoV-P=22**	**p value NP vs HP**	**p value NP vs CoV-P**	**p value HP vs CoV-P**	**ANOVA**
Age (years)	36.5 [31, 41]	33 [30.5, 34.5]	32 [27.5, 36.5]	0.0898	0.0667	0.7901	0.1201
BMI	24.45±3.13	23.68±3.1	23.52±3.76	0.3764	0.444	0.9077	0.6414
Vaccinated	16 (100%)	16 (100%)	7 (32%)	1	**<0.0001**	**<0.0001**	**<0.0001**
Gravidity (number pregnancies)	1.5 [0,3]	2 [1, 2.5]	3 [1,5]	0.4725	**0.0375**	**0.0488**	0.0955
Parity (number of deliveries)	1.5 [0,3]	2 [0, 2]	2 [0,2]	0.2807	0.6073	0.0631	0.21
Gestational week	N/A	39 [39, 40]	36 [28.5,38]	N/A	N/A	**0.0002**	N/A

Systolic blood pressure (mmHg)	112±5.1	114.9±7.26	116.6±11.74	0.3425	**0.2356**	0.3593	0.6458
Diastolic blood pressure (mmHg)	69.25±4.35	67.5±8.12	68.52±8.909	0.58	**1**	0.7116	0.8505
Heart rate (bpm)	75.2±5.40	82.21±10.36	90.2±22.17	0.146	0.051	0.189	0.12
WBC (10^3/mL)	6.57±1.08	9.881±2.02	8.564±2.201	**<0.0001**	**0.0082**	0.0625	**0.0002**
PLT (10^3/mL)	259.4±63.7	198.5±45.64	168.7±81.86	**0.0305**	**0.0117**	0.7104	**0.0279**
Fibrinogen (mg/dL)	311.4±54.57	504±92.09	527.2±95.56	**0.0003**	**<0.0001**	0.5556	**<0.0001**
D-Dimer (mg/L)	0.356±0.32	1.691±0.76	1.75±0.8593	**0.0002**	**<0.0001**	1.691	**<0.0001**

Figure 6

**FACS (CD235 CD62p)**	**NP=10**	**HP=16**	**CoV-P=22**	**p value NP vs HP**	**p value NP vs CoV-P**	**p value HP vs CoV-P**	**ANOVA**
Age (years)	35 [30.25, 36.75]	33 [30.5, 34.5]	32 [27.5, 36.5]	0.4068	0.4005	0.7901	0.6296
BMI	23.48±3.026	23.842±2.986	23.52±3.76	0.9756	0.8944	0.9077	0.9838
Vaccinated	10 (100%)	16 (100%)	7 (32%)	1	**<0.0001**	**<0.0001**	**<0.0001**
Gravidity (number pregnancies)	1.5 [0, 2.75]	2 [1, 2.5]	3 [1,5]	0.2964	**0.0489**	**0.0488**	0.0709
Parity (number of deliveries)	1 [0,2]	2 [0, 2]	2 [0,2]	0.41	0.5536	0.0631	0.514
Gestational week	N/A	39 [39, 40]	36 [28.5,38]	N/A	N/A	**0.0002**	N/A
Systolic blood pressure (mmHg)	119.6±9.76	114.9±7.26	116.6±11.74	0.2416	0.2356	0.6829	0.4463
Diastolic blood pressure (mmHg)	74.71±4.35	67.5±8.12	68.52±8.909	0.1988	1	0.1323	0.8505
Heart rate (bpm)	73±5.40	82.21±10.36	90.2±22.17	0.3018	0.044	0.189	0.0521
WBC (10^3/mL)	6.613±1.21	9.881±2.02	8.564±2.201	**0.0004**	**0.0215**	0.0625	**0.0014**
PLT (10^3/mL)	259.9±55.48	198.5±45.64	168.7±81.86	**0.0238**	**0.0099**	0.7104	**0.0244**
Fibrinogen (mg/dL)	311.4±54.57	504±92.09	527.2±95.56	**0.0008**	**0.0001**	0.5556	**0.0003**
D-Dimer (mg/L)	0.2763±0.22	1.691±0.76	1.75±0.8593	**0.0002**	**0.0001**	1.691	**0.0001**

Figure 6

**TF activity**	**NP=6**	**HP=7**	**CoV-P=16**	**p value NP vs HP**	**p value NP vs CoV-P**	**p value HP vs CoV-P**	**ANOVA**
Age (years)	35.5 [30.75, 37.75]	31 [28, 33]	30.5 [27, 36.75]	0.173	0.2518	0.9733	0.3685
BMI	23.77±3.12	23.4±2.85	24.05±4.05	0.7308	0.8153	0.6217	0.8383
Vaccinated	6 (100%)	7 (100%)	5 (31.25%)	1	0.0007	0.0007	0.0007

Gravidity (number pregnancies)	1.5 [0, 3.25]	3 [1, 5]	3 [1, 5]	0.1936	0.1604	1	0.2891
Parity (number of deliveries)	1 [0, 2.25]	1 [0.75, 2]	1.5 [0, 2.75]	0.8217	0.6707	0.8918	0.9006
Gestational week	N/A	39 [39,39]	36 [30.25, 38.5]	N/A	N/A	**0.0306**	N/A
Systolic blood pressure (mmHg)	119.2±8.09	117.1±6.96	115.4±12.81	0.7745	0.6306	0.8145	0.853
Diastolic blood pressure (mmHg)	73.67±8.57	66.86±9.72	68.13±9.77	0.224	0.3016	0.8145	0.4267
Heart rate (bpm)	77.33±9.67	84.43±10.97	92.73±18.46	0.2234	**0.0354**	0.2584	0.0698
WBC (10^3/mL)	7.083±1.12	8.957±1.21	8.48±1.94	**0.0183**	**0.0471**	0.5022	**0.0416**
PLT (10^3/mL)	286.7±64.28	196.4±55.5	194.4±47.53	**0.035**	**0.011**	0.7637	**0.0257**
Fibrinogen (mg/dL)	309.4±70.35	456±111.7	522.3±83.74	**0.035**	**0.0008**	0.2463	**0.0025**
D-Dimer (mg/L)	0.4871±0.73	1.523±0.53	1.877±0.86	**0.0248**	**0.0034**	0.426	**0.0066**
