# Supplementary material for: Characterization of extracellular vesicles in COVID-19 infection during pregnancy
Source: Front Cell Dev Biol. 2023 Jul 25;11:1135821. doi: 10.3389/fcell.2023.1135821 (PMC10407400; doi:10.3389/fcell.2023.1135821)
Supplement: Supplementary file 2 [file DataSheet4.pdf]

Contribution to the field (06 Jul 2023)

Our study characterized EVs released during COVID-19 infection in pregnant women (CoV-P) compared to healthy non-pregnant (NP) and healthy pregnant women (HP). Most CoV-P (90%) displayed mild disease without requiring oxygen administration, ICU admission, or emergent delivery due to maternal instability.

EV concentrations were higher in HP compared to NP but decreased in CoV-P patients. EVs exosome markers were higher in both HP and CoV-P-EVs compared to NP-EVs. IL-2 and IL-6 were higher in HP-EVs compared to NP-EVs but lower in CoV-P-EVs. Tissue factor levels were similar across study cohorts, whereas D-dimer and fibrinogen increased in HP and COV-P compared to NP.

Our findings suggest that CoV-P-EVs display similar coagulability characteristics as HP-EVs, but exhibit attenuated inflammation, possibly reflecting the "suppressive" impact of placental-EVs and that facilitate a favorable disease course in COVID-infected pregnant women. This study may have broader implications regarding placental EVs with immune suppressive properties and their effects on pregnant women with comorbid immunologic and infectious conditions.
