# Supplementary figures and images for: Characterization of extracellular vesicles in COVID-19 infection during pregnancy
Source: Front Cell Dev Biol. 2023 Jul 25;11:1135821. doi: 10.3389/fcell.2023.1135821 (PMC10407400; doi:10.3389/fcell.2023.1135821)

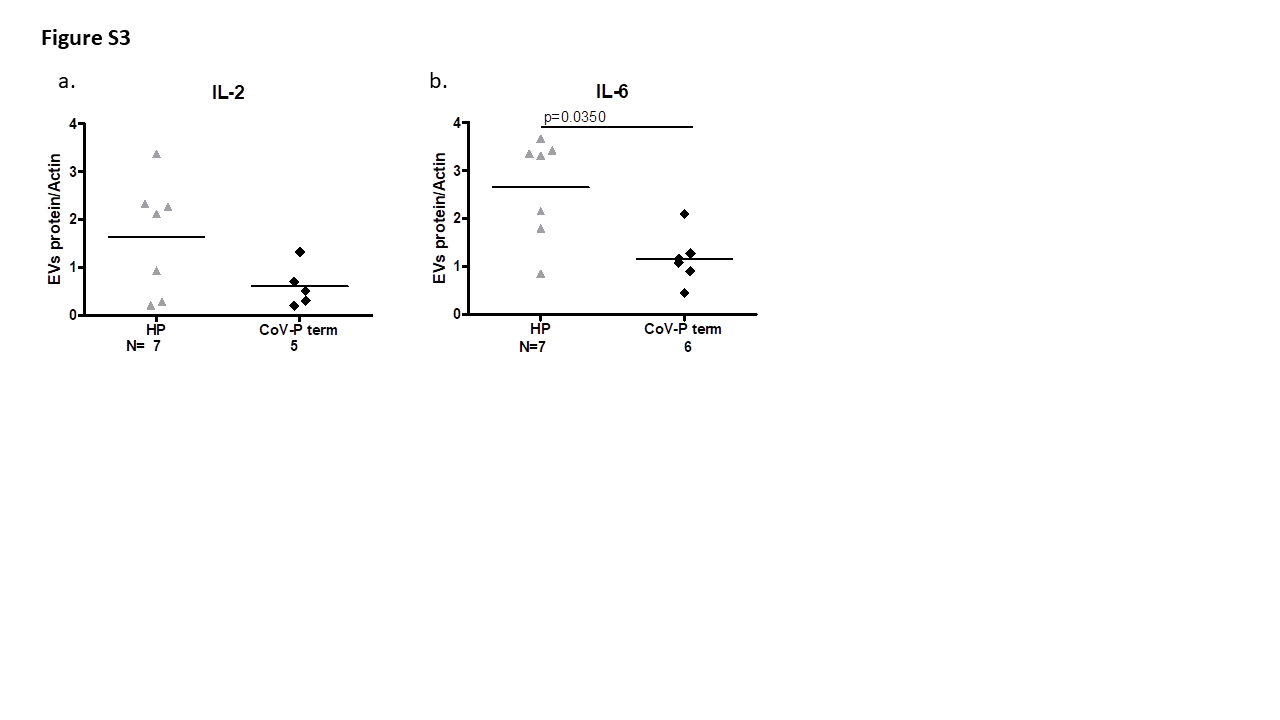

Supplement: Supplementary file 3 [file Image3.TIF]

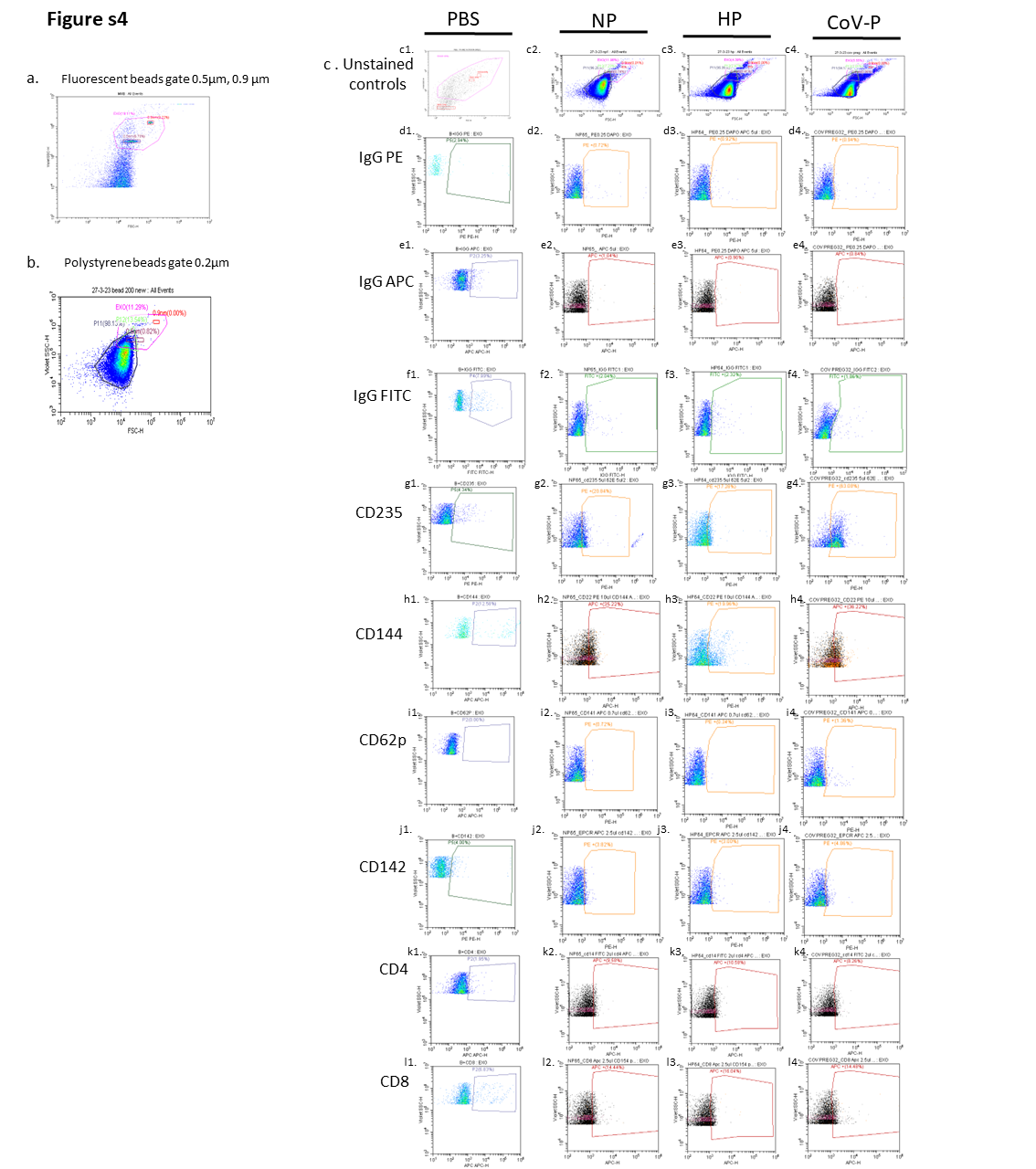

Supplement: Supplementary file 4 [file Image4.TIF]

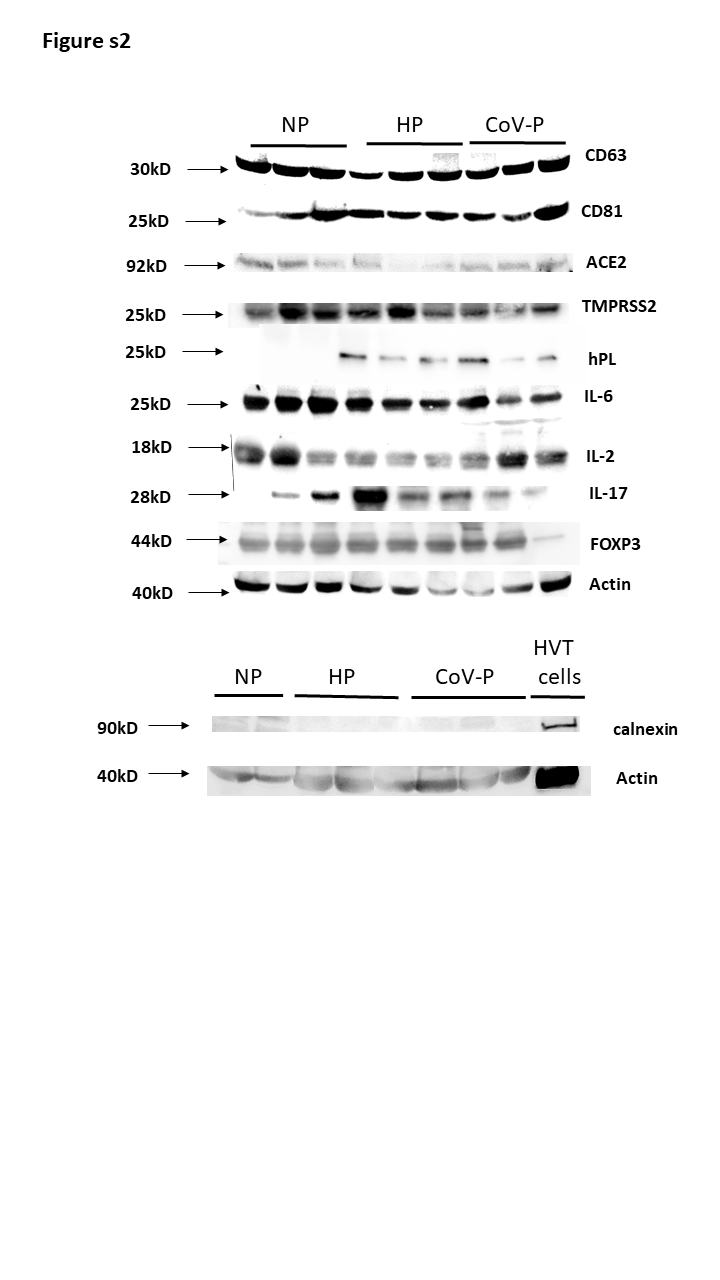

Supplement: Supplementary file 5 [file Image2.TIF]

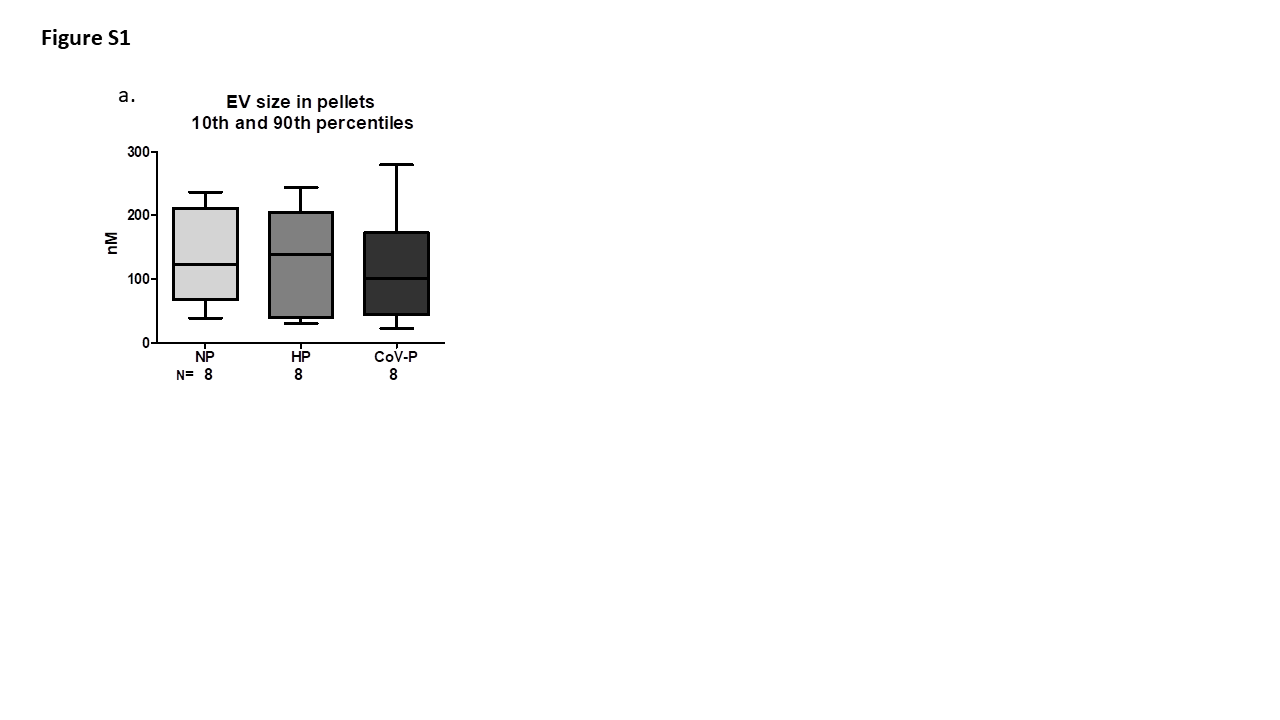

Supplement: Supplementary file 6 [file Image1.TIF]
