# Supplementary material for: Characterization of extracellular vesicles in COVID-19 infection during pregnancy
Source: Front Cell Dev Biol. 2023 Jul 25;11:1135821. doi: 10.3389/fcell.2023.1135821 (PMC10407400; doi:10.3389/fcell.2023.1135821)
Supplement: Supplementary file 7 [file DataSheet3.pdf]

Variable	Spearman r	p value
size (nM)	0.129	0.460
concentration (Evs/ml)	-0.090	0.606
Exosome marker (CD63)	0.158	0.519
Exosome marker (CD81)	0.135	0.607
hPL	0.118	0.664
TMPRSS*	-0.133	0.566
ACE2	-0.167	0.414
IL-2**	0.308	0.200
IL-6	0.079	0.742
EVs T cytotoxic marker (CD8)	-0.096	0.573
EVs T helper marker (CD4)	-0.159	0.347
Evs red blood cell marker (CD235)	-0.049	0.774
EVs Vascular-endothelial cell adhesion molecule marker (CD144)	-0.199	0.238
EVs Platelet marker(CD62p)	-0.140	0.410
EVs Tissue Factor (CD142)	-0.087	0.608

Supplementary Table S6 (results section): Correlation between pregnancy week and EV characteristics

Spearman's r correlation between pregnancy week and EV membrane antigen expression or protein expression. All comparisons are of more than 20 pairs except for TMPRSS* 18 Pairs, IL-2** 19 pairs.
