# Supplementary material for: Characterization of extracellular vesicles in COVID-19 infection during pregnancy
Source: Front Cell Dev Biol. 2023 Jul 25;11:1135821. doi: 10.3389/fcell.2023.1135821 (PMC10407400; doi:10.3389/fcell.2023.1135821)
Supplement: Supplementary file 8 [file DataSheet1.pdf]

Supplemental method information : MIFlowCyt checklist (39)

1. Preanalytical variables conforming to MISEV guidelines: EVs were isolated from platelet poor plasma (PPP) according to MISEV2018 (39). Specifically, platelet-poor plasma (PPP) was obtained after two sequential centrifugations (15 min 1500×g, 24°C) within one hour of collection and frozen in aliquots at −80°C (33). Flow cytometer analysis was performed on thawed PPP samples.

2.1 Sample staining details*: Single antibodies were added to 50µl of PPP (diluted 1:5) for 30 minutes of incubation at room temperature in the dark. Antibody dilutions are detailed in the Supplementary table S1 - antibodies list.

2.2 Sample washing details*: 400µl of filtered PBS was added to each tube at the end of antibody incubation without washing.

2.3 Sample dilution details*: PPP was diluted by 5 with filtered PBS.

3.1 Buffer-only controls* were performed and representative graph of PBS only is presented in the supplementary figure S4 –c1.

3.2 Buffer with reagent controls* were performed. EV gates were set using Megamix, a mix of fluorescent beads (0.5/0.9/3µm beads; Biocytex, Marseille, France) (Fig S4a), "gold standard" size gate calibration beads (37,38) and 0.2 µm polystyrene beads (Malvern Pananalytical, UK) (Fig S4b). Representative graphs of each antibody with PBS are presented in the supplementary figure S4 d1-L1.

3.3 Unstained controls* were performed and representative graphs appear in the supplementary figure S4 c2-c4.

3.4 Isotype controls** were performed and representative graphs appear in the supplementary figure S4 d1-4; e1-e4; f1-f4

3.5 Single-stained controls* were performed and representative graphs appear in the supplementary figure S4

3.6 Procedural controls** CytoFLEX Daily QC Fluorospheres (Cat. B53230, Beckman Coulter, Brea, CA, USA) were used as routine checklist for operation of the flow cytometer CytoFLEX, (Beckman Coulter, USA). The CytoFLEX Daily QC fluorospheres consists of a suspension of ~3 µm fluorospheres with a fluorescence emission of 410 nm to 800 nm when excited at 405 nm, 488 nm or 635 nm. CytoFLEX Daily QC Fluorospheres used for daily verification of the CytoFLEX flow cytometer's optical alignment and fluidics system.

3.7 Serial dilutions* were performed to calibrate antibody concentrations.

3.8 Detergent-treated controls were not performed.

4.1 Trigger channel(s) and threshold(s)* NA

4.2 Flow rate/volumetric quantification* - A flow rate of 10 μL per minute.

4.3 Fluorescence calibration* Violet SSC 40; PE 120; APC400; FITC 100.

4.4 Scatter calibration: Gain: FSC 500; SSC 100.

5.1 EV diameter/surface area/volume- NA.

5.2 EV refractive index approximation- NA.

5.3 EV epitope number approximation- NA.

6.1 Completion of MIFlowCyt checklist* Yes.

6.2 Calibrated channel detection range – as performed by the CytoFLEX Daily QC Fluorospheres: Gain of: FSC-74; SSC -68; FITC-129; PE-133; APC-407

6.3 EV number/concentration: ~22,000 event/μl.

6.4 EV brightness- NA.

7.1 Sharing of data to a public repository- NA.

*Highlights the components that are broadly applicable to the majority of single-EV analysis experiments regardless of design or instrumentation. No single-EV analysis experiments were performed.

**Highlights the components that are only applicable in cases where certain reagents or protocols are used.

**Antibodies and kit for western blot**	**Concentration**	**Vender**	**Cat. Number**
Mouse anti human CD63	1:1000	abcam	ab59479
Mouse anti human CD81	1:1000	abcam	ab79559
Anti-interferon gamma (IFNγ)	1:1000	abcam	ab133566
Rabbit anti human TNF alpha	1:1000	abcam	ab215188
Rabbit anti human IL-2	1:1000	abcam	ab9238
Mouse anti human IL-6	1:500	abcam	ab9324
Rabbit anti human IL-17A	1:1000	abcam	ab79056
Rabbit anti human Placental lactogen	1:25000	abcam	ab137099
Rabbit anti human TGFβ	1:1000	abcam	ab215715
Mouse anti human Actin	1:1000	Biomedical	691001MP

Rabbit anti Calnexin	1:5000	abcam	ab10286
Rabbit anti FOXP3	1:1000	abcam	ab4728
Anti-mouse	1:5000	Jackson	115-035-146
Anti-rabbit	1:5000	Jackson	111-035-144
**Antibodies for Flow Cytometry (mouse anti human)**	**Color**	**Vender**	**Cat. Number**
Mouse IgG1 isotype control	FITC	BD	345815
Mouse IgG1 isotype control	PE	BD	400112
Mouse IgG1 isotype control	APC	BD	555751
Anti CD235a (red blood cells)	PE	Dako	R7078
Anti CD41a (platelet)	APC	BD	559777
Anti CD62P (activated platelet)	PE	BD	555524
Anti CD62E (Endothelial cells)	APC	BD	551144
Anti CD144 (Endothelial cells)	APC	Biolegend	B259463
Anti CD31 (Endothelial cells & platelet)	FITC	BD	555445
Anti CD201 (Endothelial protein C receptor (EPCR), coagulation	APC	BD	563622
Anti CD142 (Tissue Factor, coagulation)	PE	BD	550312
Anti CD141 (Thrombomodulin, coagulation)	APC	BD	564123
Anti HLADR (MHC class II)	APC	BD	559868
Anti CD11a (mediate leukocyte adhesion)	PE	BD	555384
Anti CD14 (monocyte, Inflammation)	FITC-FL1	BD	555397
Anti CD4 (T-cells)	APC	BD	555349
Anti CD8 (T-cell receptor (TCR))	APC	BD	566852
Anti CD28 (T cells activation)	APC	BD	559770
Anti CD22, B lymphocyte-specific adhesion molecule	PE	BD	337899

Supplementary table S1 (method section) antibodies list

	Vaccinated CoV-P subjects	Non-vaccinated CoV-P subjects	p-value
Fibrinogen	507.4±106.5 (7)	543.2±80.6 (15)	0.4323
PLT	194.9±40.4 (7)	205.3±58.6 (15)	0.859
WBC	8.68±2.2 (7)	7.99±2.0 (15)	0.5458
D-Dimer	1.84±0.97 (7)	1.69±0.8 (15)	0.8148
EVs size	94.65±16.53 (6)	102.6±7.025 (12)	0.1632
EVs concentration	2.69E+11±1.13E+11 (6)	2.9E+11±9.8E+10 (12)	0.5262
%EVs<150nM	87.3±6.78 (6)	86.48±2.65 (12)	0.2539
CD63	0.33±0.3 (3)	0.34±0.1 (9)	0.3677
CD81	0.43±0.5 (2)	1.23±0.8 (8)	0.0727
TMPRSS	1.21±1.3 (3)	1.33±1.4 (8)	0.9307
ACE2	0.89±1.1 (5)	0.71±0.5 (12)	0.9433
hPL	1.23±0.9 (4)	0.21±0.1 (10)	0.0519
IL2	0.37±0.16 (3)	0.65±0.5 (8)	0.4286
IL6	1.58±0.7 (3)	0.99±0.43 (10)	0.1274
IL17	0.8±0.4 (2)	0.37±0.12 (8)	0.0667
TNFα	3.8±2.8 (4)	1.73±1.8 (12)	0.1806
IFNγ	2.47±1.4 (4)	2.019±2.7 (4)	0.1806
Monocytes (CD14)	5.47±8.3 (7)	0.7433±1.5 (15)	0.3297
Leukocytes (CD11a)	5.22±3.8 (7)	4.63±4.5 (15)	0.241
B cell receptor (CD22)	13.69±7.3 (7)	15.62±11.6 (15)	0.9717
HLA-DR	15.81±11.4 (7)	12.81±7.8 (15)	0.5224
EVs T cytotoxic (CD8)	19.48±10.6 (7)	15.98±5.2 (15)	0.5941
EVs T helper (CD4)	10.55±7.9 (7)	4.83±2.4 (15)	0.0361
EVs T cell co-stimulatory signal (CD28)	4.92±4.1 (7)	3.15±2.7 (15)	0.3744
EVs RBC (CD235)	31.34±13.6 (7)	29.83±8 (15)	0.6441
EVs EC adhesion molecule (CD144)	13.01±13.7 (7)	7.0±6.1 (15)	0.5458
EVs activated Platelet (CD62p)	1.82±1.9 (7)	1.85±1.6 (15)	0.6698
EVs Tissue Factor (CD142)	8.27±5.7 (7)	9.8±8.8 (15)	0.9717
EVs TF activity (pM)	47.9±8.8 (5)	72.09±50.7 (11)	0.4079

Supplementary table S2: analysis of EVs characteristics in vaccinated and non-vaccinated CoV-P subjects. Results displayed as mean ± STD. all variables were tested for normality by Shapiro-Wilk

normality test and did not show normal distribution. P-value calculated via Mann-Whitney t-test. Number of subjects in each comparison is in parenthesis. Comparisons in this table may be underpowered to detect significant differences between the groups, due to a small sample size.

Variable	**NP**	**HP**	**CoV-P**	**p value NP vs HP**	**p value NP vs CoV-P**	**p value HP vs CoV-P**	**ANOVA**
IL-2	1.06±0.52	1.58±1.12	0.52±0.38	0.4136	0.05	0.0431	0.0432
IL-6	1.19±0.91	2.41±1.2	1.22±0.77	0.036	1	0.039	0.0572
IL-17	0.7±0.9	0.9±0.94	0.54±0.38	0.5358	0.9368	0.7445	0.8369
TNFα	1.72±1.04	2.6±1.88	3.04±2.66	0.3401	0.3218	0.9323	0.5116
IFNγ	2.45±2.07	2.65±2.22	2.3±1.91	0.9314	0.6304	0.7989	0.8896
TGFβ	0.56±0.44	0.89±0.9	0.74±0.51	0.6965	0.4025	0.9711	0.7152
FOXP3 T reg	1.37±0.91	1.9±1.7	1.34±0.57	0.7304	0.8148	0.743	0.8842

Supplementary table S3 (results section): EV Cytokine content (western blot, EVs protein /Actin). Results displayed as mean ± STD. All variables were tested for normality by Shapiro-Wilk normality test and did not show normal distribution.

P-value calculated via Mann-Whitney t-test to compare two groups or Kruskal- Wallis ANOVA to compare all groups.

	**NP**	**HP**	**CoV-P**
Nano tracking analysis (NTA):			
PPP	10	16	18
EVs pellet	8	8	8
Western blot analysis:			
CD63	11	8	12
CD81	6	9	11
hPL	11	9	14
ACE2	9	14	17
TMPRs	6	7	11
IL-2	6	8	11
IL-6	6	8	13
Flow Cytometry analysis:			
CD235	9	16	21

CD144	6	16	22
CD62P	10	16	22
CD142	16	16	22
TF activity	6	7	16

Supplementary table S4 (method section) sample size in each experiment
